# Magnetization Signature of Topological Surface States in a Non‐Symmorphic Superconductor

**DOI:** 10.1002/adma.202103257

**Published:** 2021-08-08

**Authors:** Wenjun Kuang, Guillermo Lopez‐Polin, Hyungjun Lee, Francisco Guinea, George Whitehead, Ivan Timokhin, Alexey I. Berdyugin, Roshan Krishna Kumar, Oleg V. Yazyev, Niels Walet, Alessandro Principi, Andre K. Geim, Irina V. Grigorieva

**Affiliations:** ^1^ Department of Physics and Astronomy University of Manchester Manchester M13 9PL UK; ^2^ Institute of Physics Ecole Polytechnique Fédérale de Lausanne (EPFL) Lausanne CH‐1015 Switzerland; ^3^ Department of Chemistry University of Manchester Manchester M13 9PL UK; ^4^ National Graphene Institute University of Manchester Manchester M13 9PL UK; ^5^ Present address: National Innovation Institute of Defense Technology AMS Beijing 100071 China; ^6^ Present address: Instituto de Ciencia de Materiales de Madrid (ICMM) Madrid 28049 Spain

**Keywords:** magnetization and magnetic susceptibility, non‐symmorphic crystal symmetries, superconductivity, topological surface states

## Abstract

Superconductors with nontrivial band structure topology represent a class of materials with unconventional and potentially useful properties. Recent years have seen much success in creating artificial hybrid structures exhibiting the main characteristics of 2D topological superconductors. Yet, bulk materials known to combine inherent superconductivity with nontrivial topology remain scarce, largely because distinguishing their central characteristic—the topological surface states—has proved challenging due to a dominant contribution from the superconducting bulk. In this work, a highly anomalous behavior of surface superconductivity in topologically nontrivial 3D superconductor In_2_Bi, where the surface states result from its nontrivial band structure, itself a consequence of the non‐symmorphic crystal symmetry and strong spin–orbit coupling, is reported. In contrast to smoothly decreasing diamagnetic susceptibility above the bulk critical field, *H*_c2_, as seen in conventional superconductors, a near‐perfect, Meissner‐like screening of low‐frequency magnetic fields well above *H*_c2_ is observed. The enhanced diamagnetism disappears at a new phase transition close to the critical field of surface superconductivity, *H*_c3_. Using theoretical modeling, the anomalous screening is shown to be consistent with modification of surface superconductivity by the topological surface states. The possibility of detecting signatures of the surface states using macroscopic magnetization provides a new tool for the discovery and identification of topological superconductors.

## Graphical abstract

Highly anomalous surface superconductivity is reported in a non‐symmorphic superconductor, In_2_Bi, where the nontrivial band structure results in the existence of topologically protected surface states (TSS). The near‐perfect screening of low‐frequency magnetic fields well above the upper critical field is explained by proximity superconductivity of TSS. Detecting TSS via macroscopic magnetization provides a new tool for identification of topological superconductors.

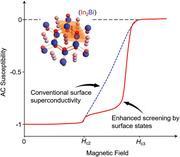

## 1 Introduction

The study of materials with nontrivial topology, including topological insulators, semimetals, and superconductors, is at the center of current condensed matter physics research.^[^ ^1^, ^2^, ^3^, ^4^, ^5^, ^6^, ^7^, ^8^, ^9^, ^10^, ^11^, ^12^, ^13^, ^14^ ^]^ The recently developed theory of topological quantum chemistry^[^ ^12^, ^13^, ^14^ ^]^ revealed that as many as a quarter of materials found in nature could possess nontrivial topology. This contrasts with the present status of experiment where relatively few materials—especially, among metals and superconductors—have been found to display tell‐tale signs of nontrivial topology, that is, topology‐protected surface states.^[^ ^3^, ^4^, ^5^, ^6^, ^15^ ^]^ Such states are particularly difficult to identify in metallic systems because their macroscopic properties are dominated by the bulk and the surface states’ contribution is often negligible. This partially explains why only surface‐sensitive techniques, such as angle‐resolved photoemission spectroscopy^[^ ^3^, ^4^, ^15^ ^]^ and tunneling spectroscopy,^[^ ^5^, ^6^ ^]^ have been successful so far in detection of topological surface states in (semi)metals and superconductors.

**Figure 1:**
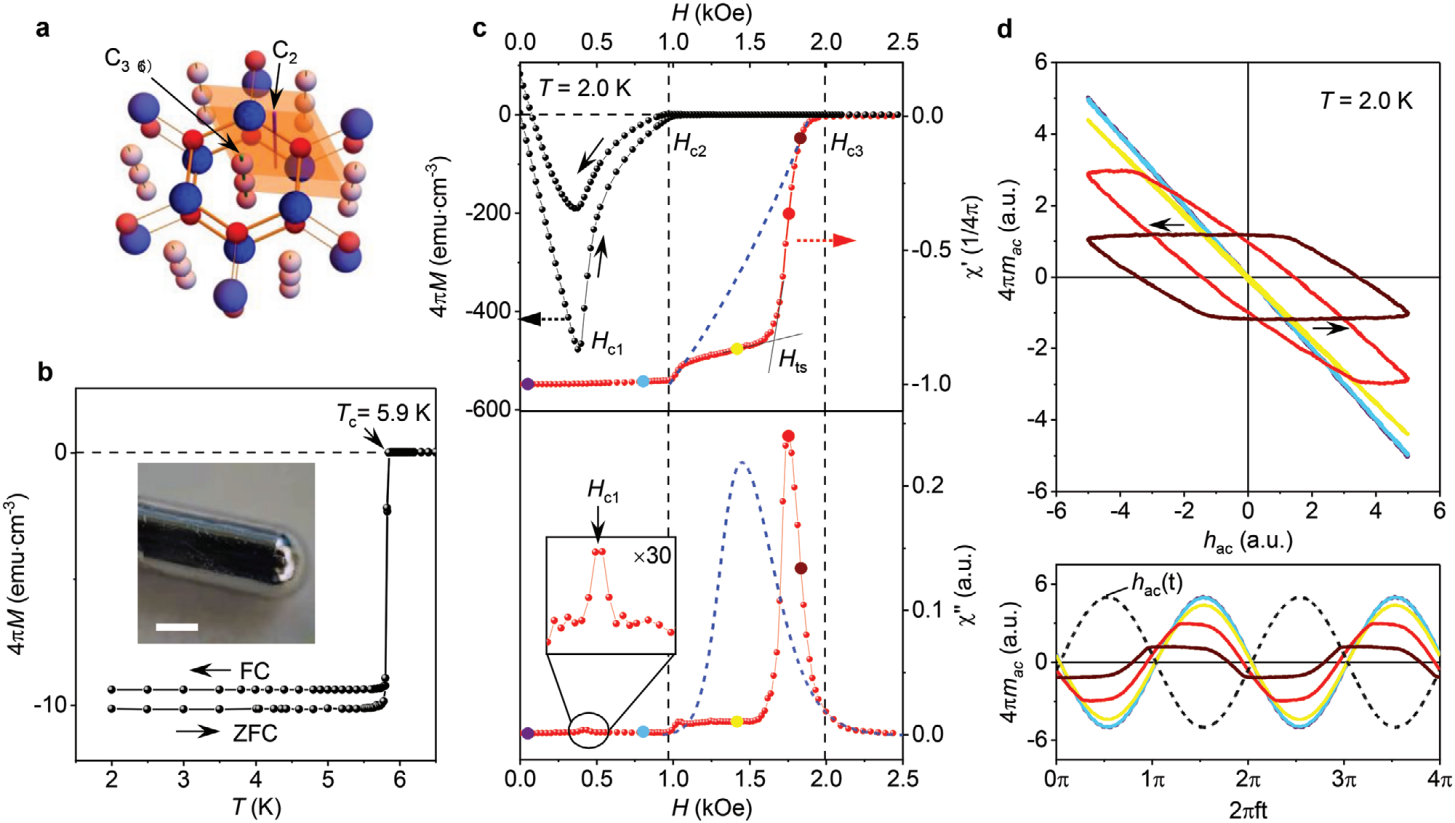
Anomalous AC susceptibility of In_2_Bi. a) Schematic crystal structure of In_2_Bi. Bi atoms are shown in blue and In atoms in different shades of red, to distinguish between In atoms within the hexagonal planes (dark red) and those making up In chains (light red). The shaded areas denote the unit cell containing four In and two Bi atoms. Symmetry axes are indicated by arrows. b) ZFC and FC magnetization as a function of *T* at *H* = 10 Oe. Inset: photo of our typical cylindrical crystal; scale bar: 1 mm. c) AC susceptibility measured using *h*_ac_ = 0.1 Oe and frequency *f* = 8 Hz (red curves). Black curves: DC magnetization and its hysteresis for this sample. As a reference, the blue dashed curves show the standard response expected for surface superconductivity. The inset in the lower panel shows a zoom of χ′′ indicating the transition to the vortex state at *H*_c1_. The vertical dashed lines indicate *H*_c2_ and *H*_c3_, and the arrows the sweep directions. d) Top: Lissajous loops for the representative DC fields indicated by the color‐coded dots in (c). Bottom: Corresponding waveforms *m*_ac_(*t*) for the applied sinusoidal field *h*_ac_(*t*) of amplitude 0.1 Oe.

Here, we use an intrinsic superconductor In_2_Bi to demonstrate that topological surface states have a distinct signature in surface magnetization and can be detected above the bulk critical field for superconductivity, *H*_c2_. The existence of surface states is predicted as a result of this material's nontrivial band structure, which itself is a consequence of the non‐symmorphic crystal symmetry (implied by the space group *P*6_3_/*mmc*) and strong spin–orbit coupling due to large atomic numbers of the constituent elements. The crystal structure of In_2_Bi is illustrated in **Figure** **1**a and can be viewed as a combination of two interpenetrating crystal lattices: a layered arrangement of In–Bi planes forming a hexagonal lattice in each layer (below we refer to these as In_1_Bi_1_) and a triangular array of 1D chains of In atoms piercing the centers of In_1_Bi_1_ hexagons. The screw symmetry of In_2_Bi is associated with the AA′ stacked monolayers of In_1_Bi_1_ whereas the In chains give the crystal its 3D character and presumably ensure little anisotropy in this material's properties.^[^ ^16^, ^17^, ^18^ ^]^ In fact, the crystal symmetry of In_2_Bi is similar to that of several known topological materials, including Dirac semimetal Na_3_Bi,^[^ ^15^ ^]^ non‐symmorphic topological insulator KHgSb,^[^ ^19^ ^]^ and heavy‐fermion odd‐parity superconductor UPt_3_^[^ ^20^ ^]^ where the surface states have been either predicted by theory or observed using surface‐science techniques. Although basic superconducting characteristics of In_2_Bi have been known for decades,^[^ ^16^, ^17^, ^18^ ^]^ no attention had been paid to the nontrivial crystal symmetry and its consequences for the nature of superconductivity.

## 2 Results and Discussion

Importantly for the present study, we have succeeded in growing high‐quality single crystals of In_2_Bi, as confirmed by X‐ray diffraction analysis (for details of the crystal growth and characterization, see Experimental Section and Figures S1 and S2, Supporting Information). Both spherical and cylindrical samples of ≈2 mm in diameter, *d*, were studied, with all crystals exhibiting smooth, mirror‐like surfaces (insets of Figure 1b and Figure S3, Supporting Information). Below we focus on the results obtained for cylinders because of the simple geometry, best suitable for magnetization studies (spherical samples exhibited essentially the same behavior described in the Supporting Information). The high quality of our In_2_Bi samples is also evident from the sharp (<0.1 K) superconducting transition at *T*_c_ = 5.9 K, little hysteresis between zero‐field cooling (ZFC) and field‐cooling (FC) magnetization (Figure 1b), and nearly absent remnant magnetization *M* (Figure 1c) indicating little flux trapping (pinning). The well‐defined demagnetization factor for our crystals’ geometry allowed us to accurately determine the characteristic parameters of In_2_Bi superconductivity using the DC magnetization curves *M*(*H*), such as shown in Figure 1c and Figure S3, Supporting Information. At *T* = 2 K (our lowest measurement temperature), we found the lower and upper critical fields *H*_c1_ ≈  490 Oe and *H*_c2_ ≈  950 Oe, respectively, coherence length ξ ≈ 60 nm, magnetic field penetration depth λ ≈ 65 nm, and the Ginzburg–Landau (GL) parameter, κ = λ/ξ, close to 1 (but see further for the temperature dependence of κ). In terms of these key superconducting parameters, In_2_Bi is similar to very pure Nb,^[^ ^21^, ^22^ ^]^ one of the most‐studied low‐κ superconductors, which allows comparison with conventional behavior.

Figure 1c,d presents our central observations: an anomalous magnetic response above the bulk critical field *H*_c2_, where superconductivity is retained within a thin surface sheath of thickness ≈2ξ^[^ ^23^, ^24^ ^]^ and exists up to the critical field for surface superconductivity, *H*_c3_. Surface superconductivity in conventional superconductors had been studied in much detail in the past, both theoretically and experimentally, and is known to have specific signatures in AC susceptibility χ = χ′ + iχ′′ and DC magnetization *M*(*H*) above *H*_c2_. The contribution of the surface superconducting sheath to magnetization is particularly significant for pure superconductors with λ ≈ ξ,^[^ ^24^, ^25^, ^26^, ^27^ ^]^ as in our case. As detailed in Supporting Information (“AC susceptibility and DC magnetization in conventional superconductors: Contribution of surface superconductivity”), above *H*_c2_ the real part of AC susceptibility χ′ is expected to evolve smoothly, decaying approximately linearly from the full Meissner screening at *H*_c2_ to zero at *H*_c3_. At the same time, χ′′ should exhibit a broad peak between *H*_c2_ and *H*_c3_ (Figure 1c). This standard behavior has been well‐understood theoretically^[^ ^24^, ^25^, ^26^, ^27^, ^28^, ^29^ ^]^ as a consequence of shielding by the supercurrent induced in the surface sheath. The susceptibility is described by^[^ ^28^, ^29^ ^]^ χ′ = Re{[− 1 + 2*J*_1_(*Kd*)/*KdJ*_0_(*Kd*)]/4π} where *J*_0_ and *J*_1_ are Bessel functions and, ignoring the skin‐effect and the contribution from normal electrons, $K^{2}\approx -1/\lambda_{L}^{2}=-4\pi n_{s}e^{2}/mc^{2}$. Here, λ_L_ is the London penetration depth, *n*_s_∝ |Ψ|^2^ the surface superfluid density, and Ψ the order parameter.^[^ ^30^ ^]^ The decrease in χ′ with increasing applied field *H* (blue curve in Figure S10b, Supporting Information) corresponds to a reduction of *n*_s_(*H*) inside the surface sheath (Figure S10a, Supporting Information) and a corresponding increase in λ_L_, so that the screening ability of the sheath is gradually reduced. The broad maximum in χ′′ is due to normal electrons that appear above *H*_c2_ and lead to dissipation, as they are accelerated by the electric field *E* ∝ d*j*_S_/d*t* (*j*_S_ is the supercurrent density).^[^ ^31^ ^]^ Qualitatively, it can be explained as follows: as the DC field increases above *H*_c2_, *n*_S_ and *j*_S_ are sufficiently large to cause an overall increase in dissipation as the density of normal electrons *n*_n_ increases. However, as *n*_S_ decreases further closer to *H*_c3_, so does *j*_S_ and *E*, which reduces the force on the normal fluid and the dissipation (at low frequencies used in our measurements the normal‐state response is negligibly small). The expected χ′(*H*) and χ′′(*H*)—which also reproduce the behavior observed in pure conventional superconductors^[^ ^21^, ^22^ ^]^—are shown in Figure 1c by the dashed blue lines (for further details, see Supporting Information and Figure S10, Supporting Information).

In contrast to the described conventional behavior, AC susceptibility of In_2_Bi changes little above *H*_c2_, showing near‐perfect diamagnetism up to a certain, rather large, field *H*_ts_ just below *H*_c3_ (Figure 1c). There is a small decrease in χ′ but otherwise In_2_Bi exhibits a nearly complete Meissner effect with respect to the AC field. This is accompanied by vanishingly small dissipation χ′′ which indicates that the density of normal electrons remains negligibly small (see above). Only at *H*_ts_, both χ′ and χ′′ change abruptly, suggesting another phase transition, additional to the transitions at *H*_c1_, *H*_c2_, and *H*_c3_. This anomalous behavior becomes even clearer when we consider individual cycles of the magnetization, *m*_ac_(*t*), and the corresponding Lissajous loops *m*_ac_(*h*_ac_), where *h*_ac_(*t*) is the applied AC field (Figure 1d). Below *H*_ts_ (yellow curves), *m*_ac_(*h*_ac_) are linear with 180° phase difference between *m*_ac_ and *h*_ac_, which indicates dissipation‐free diamagnetic screening of the AC field. This behavior persists up to *H*_ts_ and is nearly identical to the full Meissner screening below *H*_c2_ (blue curves). Only above *H*_ts_, the AC susceptibility starts exhibiting the response normally expected for surface superconductivity: *m*_ac_ decreases and out‐of‐phase signal appears so that the sinusoidal waveforms become strongly distorted (red and brown curves in Figure 1d) while χ′(*H*) smoothly decreases to zero at *H*_c3_ and χ′′(*H*) shows a corresponding maximum (Figure 1c).

**Figure 2:**
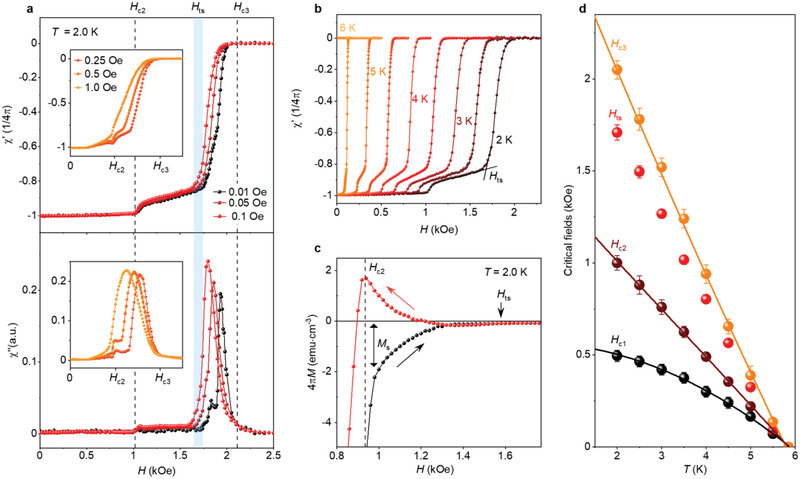
Anomalous diamagnetic response at different temperatures and AC excitations. a) AC susceptibility as a function of the AC field amplitude (see legends). b) χ′(*H*) at *T* between 2 and 6 K measured with 0.5 K step; *h*_ac_ = 0.1 Oe. c) Hysteresis in *M*(*H*) between the increasing (black symbols) and decreasing (red) DC field *H*; *T* =  2 K, *H*_ts_ is indicated by an arrow. d) Phase diagram for all the critical fields (labeled and color coded). Red symbols: *H*_ts_(*T*) found from AC susceptibility measurements in (b). Error bars: standard deviations. The black curve shows the standard BCS dependence *H*_c1_(*T*) ∝ 1 − (*T*/*T*_c_)^2^. Brown curve: best fit to *H*_c2_(*T*) using the two‐band model of superconductivity (Supporting Information). Yellow curve: guide to the eye. The *H*_c3_/*H*_c2_ ratio changes from 2.0 at 2 K to 1.7 at 5.6 K, as expected (see text).

To observe this anomalous behavior, it was essential to use very small AC fields. We could clearly see the transition at *H*_ts_ in both χ′ and χ′′ only using *h*_ac_ below 0.1 Oe (**Figure** **2**a). For larger *h*_ac_, the additional features rapidly washed out, and only the standard behavior could be seen for *h*_ac_ ≥ 1 Oe (insets of Figure 2a). The phase transition at *H*_ts_ was particularly clear for our smallest *h*_ac_ = 0.01 Oe (measurements became progressively noisier at smaller *h*_ac_) where χ′′ splits into two peaks and the shapes of χ′(*H*) and χ′′(*H*) at *H*_ts_ strongly resembled those observed near *H*_c2_ but at much larger *h*_ac_ (cf. curves for 0.01 and 1 Oe). This similarity serves as yet another indication of the new phase transition at *H*_ts_. The observed sensitivity to the excitation amplitude is not surprising, as surface superconductivity is generally characterized by small critical currents *j*_C_ and, therefore, can screen only small AC fields.^[^ ^27^ ^]^ Furthermore, we found that the transition at *H*_ts_ could be distinguished at all *T* up to 5 K ≈ 0.85*T*_c_ (Figure 2b) and became smeared at higher *T*. The observed *T* dependences for all three critical fields are shown in Figure 2d, where *H*_ts_(*T*) follows the same, almost linear, dependence as *H*_c3_ (as expected,^[^ ^32^ ^]^ the *H*_c3_/*H*_c2_ ratio is temperature dependent, with low‐*T H*_c3_/*H*_c2_ = 2.0 decreasing to 1.69 at *T*_c_, while *H*_c2_(*T*) for In_2_Bi is linear in the available *T* range; the linearity is discussed below).

**Figure 3:**
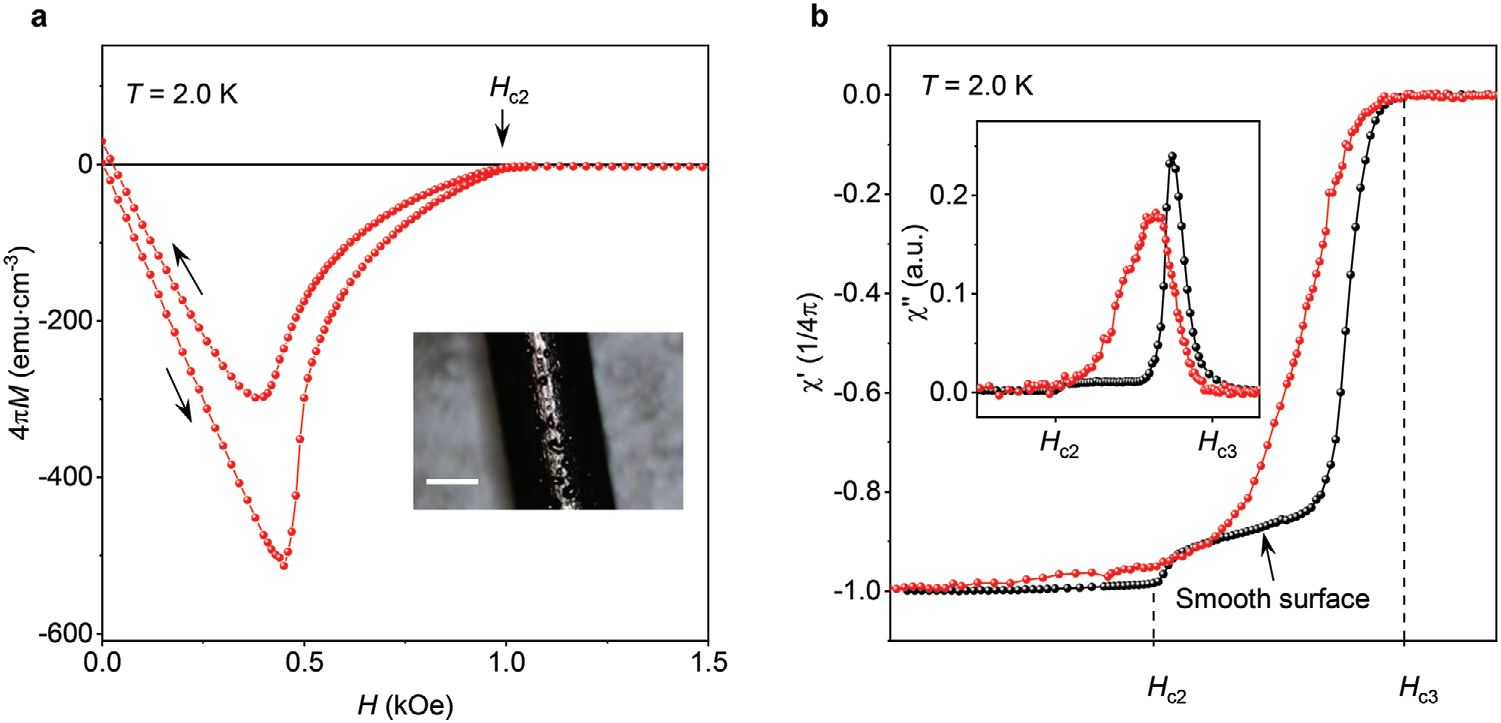
Effect of surface quality. a) DC magnetization for a sample with a rough surface shown in the photo (scale bar: 0.5 mm). Hysteresis in *M*(*H*) between increasing and decreasing field remains small, comparable to our best crystals (cf. Figure 1c). This indicates that the surface roughness did not affect the quality of the bulk. b) Comparison of AC susceptibility for crystals with comparable bulk pinning but smooth and rough surfaces (black and red curves, respectively). In both cases, *h*_ac_ = 0.1 Oe. See also Figure S3, Supporting Information, for similar data on a spherical crystal before and after intentional surface degradation.

The surface superconductivity of In_2_Bi could be discerned even in our DC magnetization measurements (Figure 2c and Figure S3b, Supporting Information), which is unusual for a bulk superconductor, even for κ ≈ 1: First, this requires the presence of a continuous sheath of supercurrent which in bulk samples is typically interrupted by “weak links” created by imperfections at the surface of realistic crystals; the weak links allow magnetic flux penetration and reduce the diamagnetic response.^[^ ^27^, ^33^ ^]^ Second, even in the ideal case, the corresponding DC signal at *H*_c2_ is only *M*_S_ ∝ (*H*_c_/κ)(λ/*R*)^1/2[^ ^25^, ^27^ ^]^ (*H*_c_ is the thermodynamic critical field and *R* radius of the cylinder). In our case 4*πM*_S_ ≈ 3G (Figure 2c), that is, corresponds to the maximum theoretical value for In_2_Bi parameters.^[^ ^25^ ^]^ The contribution is diamagnetic if *H* is increased, and paramagnetic for decreasing *H*, leading to a large hysteresis (Figure 2d). The hysteresis remained experimentally detectable in *H* close to, but below *H*_ts_. This behavior is consistent with the presence of a continuous sheath of supercurrent at the surface, which prevents the magnetic flux from entering and exiting the normal‐state bulk, leading, respectively, to a diamagnetic‐ and paramagnetic‐response.^[^ ^25^, ^26^, ^27^, ^33^ ^]^ The importance of the continuous sheath of current at the surface for the anomalous diamagnetism in our In_2_Bi is further confirmed by its sensitivity to surface quality. When we intentionally introduced surface roughness, the anomalous features below *H*_ts_ disappeared and the response became conventional with a smooth decrease of χ′, a broad peak in χ′′ (**Figure** **3** and Figure S3, Supporting Information), and no hysteresis in *M*(*H*) above *H*_c2_, even though the critical fields *H*_c1_, *H*_c2_, and *H*_c3_ were essentially unaffected. In contrast, bulk disorder was found to be less important for the anomalous behavior: bulk pinning reduced the diamagnetic susceptibility between *H*_c2_ and *H*_ts_ but the transition at *H*_ts_ can still be seen in our In_2_Bi crystals even with stronger pinning (Figure S4, Supporting Information). This further emphasizes the importance of the surface for the observed anomalous screening.

**Figure 4:**
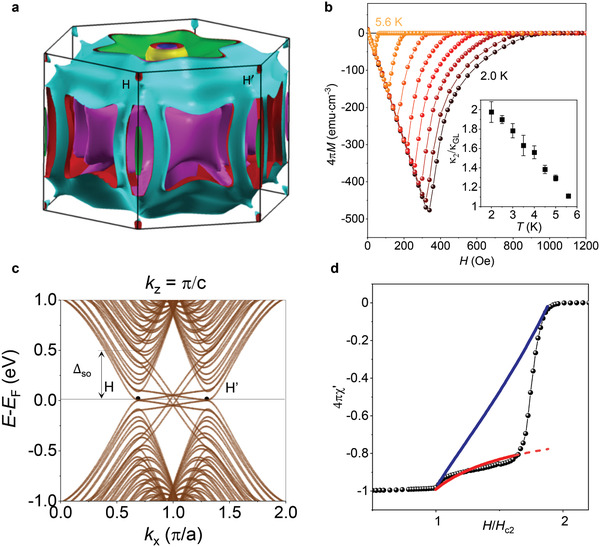
Band structure of In_2_Bi and experimental evidence of multiband superconductivity. a) Calculated Fermi surface of In_2_Bi. b) Temperature evolution of DC magnetization. The curves are for *T* = 2.0, 2.5, 3.0, 3.5, 4.0, 4.5, 5.0, and 5.6 K. Inset: Temperature dependence of the extracted Maki parameter κ_2_. Error bars: standard deviations. c) Band structure of an In_2_Bi ribbon near the H (H′) points (see “Topological surface states” in Supporting Information for details). Bands due to In chains are omitted for clarity. Four pairs of counter‐propagating edge states cross within the bulk bandgap. Two of the pairs connect bands split by the spin–orbit gap (Δ_SO_) between Bi‐derived bands, while the other two are within a smaller gap of In‐derived bands. In these calculations, hopping amplitudes that break particle–hole symmetry were not included. d) Comparison of the observed AC response (symbols) with the theory for conventional surface superconductivity (blue curve) and our model that includes proximitized surface states (red).

To explain the highly anomalous diamagnetism between *H*_c2_ and *H*_ts_, let us first consider the electronic structure of In_2_Bi. It is shown in **Figure** **4**a as calculated using ab initio density functional theory and elucidated by tight‐binding calculations (Supporting Information). Although the entire Fermi surface of In_2_Bi is extremely complex with many sheets, one can immediately see one important feature of the electronic structure. The Fermi surface consists of cylinder‐shaped parts extended along the *z*‐axis, as well as rounded pieces. The former is a result of weakly coupled In_1_Bi_1_ planes that bring a 2D character whereas the rounded parts, indicating isotropic, 3D charge carriers, arise mostly from the In chains, as mentioned in the introduction. This combination of quasi‐2D and 3D Fermi surfaces has profound implications for superconductivity and, in particular, explains the unusual linear *T* dependence of *H*_c2_ and *H*_c3_ (Figure 2d). Such behavior is in fact expected for multiband superconductivity arising simultaneously from 2D‐ and 3D‐type Fermi surfaces^[^ ^34^, ^35^ ^]^ (for details, see ‘Temperature dependence of *H*_c2_: fitting to the multiband theory’ in Supporting Information). The multiband superconductivity in In_2_Bi and the importance of the contribution from 2D In_1_Bi_1_ sheets characterized by non‐symmorphic symmetry are also corroborated by Figure 4b that shows pronounced changes in the shape of magnetization curves with increasing *T*. At low *T*, In_2_Bi exhibits *M*(*H*) typical for conventional type‐II superconductors with low *κ*, but the dependence becomes borderline type‐I closer to *T*_c_ (see the curve at 5.6 K). This can be quantified^[^ ^35^ ^]^ using the ratio of the GL parameter κ_GL_ and the Maki parameter κ_2_ obtained from the magnetization slope d*M*/d*H* close to *H*_c2_:

(1) $$\begin{array}{c} 4\pi \frac{dM}{dH}\;{|}_{H\;=H_{c2}\;}=\frac{1}{\beta_{L}\left(2\kappa_{2}^{2}-1\right)} \end{array}$$

where β_L_ = 1.16. The Maki parameter κ_2_(*T*) found from our measurements is plotted in the inset of Figure 4b. For single‐band superconductors, κ_2_(*T*) is known to vary little (<20%) with *T* so that its value remains close to κ_GL_. In our case, *κ_2_*/κ_GL_ changes by a factor of 2, which corresponds to a multiband superconductor with Fermi surfaces having different symmetries,^[^ ^34^, ^35^ ^]^ in agreement with the electronic structure of In_2_Bi.

Another essential feature found in our band structure calculations is Dirac‐like crossings near H (and H′) points in the Brillouin zone. This is shown in Figure 4c for the case of a finite width ribbon (full band diagram is provided in Figures S6 and S8, Supporting Information). The crossings are a result of the crystal symmetry of In_2_Bi, which combines a screw‐symmetry axis (C_2_) and a threefold rotational symmetry axis (C_3_) (Figure 1a). In particular, the C_2_ screw symmetry effectively decouples the electronic states within individual In_1_Bi_1_ planes (for *k_z_* = π/*c*, where *c* is the interplane distance) and provides two copies of an “asymmetric” Kane–Mele model.^[^ ^36^ ^]^ Spin–orbit interaction (strong for In_2_Bi) lifts the degeneracy of the corresponding Dirac‐like bands at H (H′) points, opening a large spin–orbit gap of about 0.5 eV, which—as is well known from the literature^[^ ^36^, ^37^, ^38^, ^39^ ^]^—hosts topological surface states for most surface terminations. Figure 4c shows representative results for a zig‐zag termination, as described in the Supporting Information (“Topological Surface States”). Our DFT calculations (Figure S6, Supporting Information) show that, in pristine In_2_Bi, the Fermi energy crosses the Dirac‐like bands near their touching point and, therefore, crosses the surface states as well. In the case of In_2_Bi, these states are confined to a few atomic layers at the surface (Figure S9, Supporting Information) and, as we show below, should have a profound effect on surface superconductivity, consistent with the experimental observations.

To evaluate the effect of the topologically protected ultrathin layer on the overall diamagnetic response, we note that the surface states are expected to couple with bulk superconductivity and also become superconducting by proximity,^[^ ^40^, ^41^ ^]^ creating an “outer‐surface” superconducting layer. Above *H*_c2_, bulk superconductivity is confined to ≈ 2ξ ≈ 120 nm‐thick macroscopic layer at the surface (below we refer to it as “standard surface superconductivity”); it is this relatively thick superconducting layer that is proximity coupled to the surface states. To account for this coupling, we have extended the standard GL description of surface superconductivity^[^ ^24^ ^]^ to include the proximitized surface states that are modeled as a superconducting film of thickness *d* << ξ, λ. As detailed in Supporting Information (“Effect of topological surface states on surface superconductivity”), this film effectively “pins” the amplitude of the order parameter at the surface to its maximum value *F* = 1 for all *H* < *H*_ts_, see Figure S11a, Supporting Information (this is to be compared with a gradual suppression of *F*(0) by *H*_c2_ < *H* < *H*_c3_ for standard surface superconductivity, Figure S11a, Supporting Information, and ref. ^[^ ^24^ ^]^). Due to coupling between this *H*‐insensitive outer sheath and the standard surface superconductivity, Cooper pairs in the overall ≈ 2ξ thick surface layer become much more robust with respect to pair‐breaking by the magnetic field, and the superfluid density *n*_s_ remains at ≈70% of its maximum value even at *H* ≈ *H*_c3_ (Figure S11b, Supporting Information). Figure 4d shows the calculated evolution of χ′(*H*) ∝ *n*_s_ between *H*_c2_ and *H*_ts_, which is different from the conventional response but in agreement with the experiment. The model also allows us to understand other features of the anomalous response below *H*_ts_. First, its exceptional sensitivity to *h*_ac_ (Figure 2a) can be related to a finite depairing current density *j*_0_ within the outer‐surface layer. Indeed, *j*_0_ is given by the thermodynamic critical field (or the superconducting gap)^[^ ^30^ ^]^ and typically is ≈10^10^ to 10^11^ A m^−2^. Because most of the screening current flows within the 1 nm‐thick outer‐layer (where *F* is maximized, Figure S11a, Supporting Information), it is straightforward to estimate that the layer can sustain only $h_{\text{ac}}\leq \;\;1\;\text{Oe}$, in good agreement with experiment. Note that the standard surface superconductivity can support similar *j*_C_ but the current flows through a much thicker (≈2ξ) layer and, therefore, should sustain proportionally larger *h*_ac_. Second, χ′′ depends on the number of normal electrons contributing to dissipation, as discussed above. For nearly constant *n*_s_ between *H*_c2_ and *H*_ts_, the corresponding χ′′ should be negligibly small compared to conventional surface superconductivity, which explains little dissipation below *H*_ts_ (Figures 1c and 2a). Finally, the transition at *H*_ts_ probably corresponds to a switch of the outer‐surface layer into the normal state. This is largely expected as the outer‐surface superconductivity is proximity‐induced and, therefore, should have a smaller gap than the intrinsic one and be destroyed at some field *H* = *H*_ts_ < *H*_c3_. Above this field, only the standard surface superconductivity provides diamagnetic screening.

We note that the above model does not invoke the topological nature of the surface states and in principle could be realized if a “conventional” atomically thin metallic layer were present at the surface. Such a trivial scenario, however, discounts two important facts: First, the strong diamagnetic response is observed in all our samples with a good degree of crystal purity, either cylindrical or spherical. This suggests that the surface metallic state responsible for modifying the behavior of |Ψ| at the surface is a robust feature of In_2_Bi and cannot be the result of, for example, trapping by some random surface potential or another artifact. Second, the Meissner‐like screening of the whole volume of our In_2_Bi crystals above *H*_c2_ requires a continuous sheath of supercurrent at the surface. This is a stringent condition that usually cannot be met in bulk superconductors due to inevitable surface imperfections.^[^ ^33^ ^]^ In topological materials surface defects—as long as they are non‐magnetic—do not cause backscattering and do not disrupt topologically protected counter‐propagating surface currents because the existence of the topological surface states depends only on the global symmetry properties of the crystal, not on local properties of the surface. This follows from the general concept of topological states and was confirmed in experiments on topological insulators, for example, in ref. ^[^ ^43^ ^]^ where the topologically protected surface‐conducting sheath was shown to envelop the entire surface of a crystal, despite rough surfaces with stacked edges, steps, and different terminations. The presence of symmetry‐protected topological surface states in In_2_Bi offers a natural explanation for the high degree of reproducibility and robustness of the continuous sheath of supercurrent. A further indication of the topological nature of the superconducting outer‐sheath in our experiments is given by the complete suppression of the anomalous diamagnetism by surface roughness (Figure 3 and Figure S3, Supporting Information). This is likely to be caused by the introduced point‐like defects (e.g., vacancies), which have been shown^[^ ^42^ ^]^ to result in localized states and interaction‐induced magnetic moments, similar to the effect of point defects in graphene. The latter introduce backscattering of the topological states and should strongly suppress their (super)conductivity.^[^ ^42^ ^]^

Finally, attributing the enhanced diamagnetism to an accidental metallic sheath at the surface contradicts our other observations, such as the effect of allowing a thin layer of In_5_Bi_3_ (*T*_c_ ≈ 4.2 K) to form at the surface of In_2_Bi (see “Evidence of In_2_Bi Oxidation in Air and the Importance of Surface Protection” in the Supporting Information). In the temperature interval between 4.2 and 5.9 K, this corresponded to enveloping superconducting In_2_Bi in a thin (sub‐micrometer) layer of normal metal. In stark contrast to our main observations in Figures 1 and 2, this resulted in an almost complete suppression of χ′ at *T* > 4K and a sharp reduction of *H*_c3_, that is, an effect opposite to the described contribution of the superconducting topological states. Neither can the observed strong diamagnetism below *H*_ts_ be explained by the standard surface superconductivity that is somehow non‐uniform, for example, due to a slightly varying stoichiometry of the crystals at the surface. First, we carefully checked the structure and chemical composition of our crystals before and after the measurements, and these remained unchanged. More importantly, non‐uniformity always leads to an increased pinning which would reduce the surface diamagnetic screening rather than enhancing it (Figure S4, Supporting Information), again in contrast to our observations.

## 3 Conclusion

The above model based on proximity‐induced superconductivity of topological surface states qualitatively explains all the main features seen experimentally. Nevertheless, a more quantitative understanding is certainly desirable, which should take into account the unconventional symmetry of the topological states’ pairing wavefunction and consider self‐consistently their coupling to bulk superconductivity, beyond the phenomenological GL theory. Independent of the microscopic mechanism, the observed enhanced surface diamagnetism can be employed to probe possible topological superconductors and, if found, our results show that effects of topological superconductivity can be isolated from the obscuring conventional contribution from the bulk by using magnetic fields above *H*_c2_.

## 4 Experimental Section

### Crystal Growth and Characterization

To grow single crystals of In_2_Bi, the approach of ref. ^[^ ^44^ ^]^ was followed, which was known to result in spontaneous formation of spherical single crystals of 1–2 mm diameter. To this end, indium (99.99% Kurt Lesker) and bismuth (99.999% Kurt Lesker) pellets were mixed in stoichiometric composition in a quartz ampoule. The ampoule was sealed and annealed under vacuum (10^−6^ mbar) at 500 °C for 24 h. The resulting alloy was remelted at 150 °C in an oxygen‐ and moisture‐free atmosphere of an argon‐filled glove box under slow rotation at 1–2 rpm for further homogenization. This resulted in spontaneous formation of spherical single crystals of ≈0.3−2 mm diameter, as reported previously.^[^ ^44^ ^]^ Following the method of ref. ^[^ ^44^ ^]^ the crystals were kept at 100 °C for further 5 min and then allowed to cool down naturally to room temperature. To grow crystals in a long cylinder geometry, several spherical single crystals were remelted in a sealed quartz tube of ≈2 mm diameter and annealed for 2 weeks under vacuum at 87 °C (just below the melting temperature of In_2_Bi, 89 °C). This produced high‐quality cylindrical crystals with smooth surfaces, such as shown in the inset of Figure 1b. All the above procedures and further handling of the crystals were carried out in the protective atmosphere of an Ar filled glovebox (O_2_ < 0.5 ppm, H_2_O < 0.5 ppm). Once grown, care was taken to avoid exposure of the crystals to air or moisture by immediately transferring them in the vacuum environment of a cryostat or immersing in paraffin oil. This was necessary to prevent oxidation of Bi at the surface, as it was found that a prolonged (few hours) exposure to ambient atmosphere led to formation of a thin surface layer of InBi and/or In_5_Bi_3_ (see “Evidence of In_2_Bi oxidation in air and the importance of surface protection”, ).

The monocrystallinity of the samples was confirmed by X‐ray diffraction that showed sharp diffraction patterns corresponding to a primitive hexagonal unit cell with *a* = 5.4728(8) Å and *c* = 6.5333(12) Å, in agreement with the literature for stoichiometric In_2_Bi. Data on spherical In_2_Bi crystals were collected in a Rigaku FR‐X DW diffractometer using Mo Kα radiation (λ = 0.71073 Å) at *T* =  150 K and processed using Rigaku CrysAlisPro software.^[^ ^45^ ^]^ Due to absorption of the diffracted beam by heavy Bi and In atoms, even the 0.3 mm diameter crystal (used to obtain XRD data in Figure S1a, Supporting Information) was still too large to collect diffraction data from the whole sample. To overcome this problem, a glancing beam going through different edges of the spherical crystal was used. First the top of the sphere was centered in the beam, then a 4‐circle AFC‐11 goniometer used to access a wide range of crystal orientations, with the center of rotation kept at the intersection between the beam and the crystal. Reorienting the crystal allowed the authors to collect all reflections that fulfill the Bragg condition. See “Structural Characterization of In_2_Bi Crystals” in the Supporting Information for further details.

### Magnetization Measurements

Magnetization measurements were carried out using a commercial SQUID magnetometer MPMS XL7 (Quantum Design). Prior to being placed in the magnetometer, samples were mounted inside a low‐magnetic background gelatine capsules or a quartz tube, taking care to protect them from exposure to air. In ZFC mode of DC magnetization measurements the sample was first cooled down to the lowest available temperature (1.8 K) in zero magnetic field, then a finite field applied and magnetization measured as a function of an increasing temperature *T*. In FC mode, the field *H* was applied above *T*_c_ (typically at 10–15 K) and magnetization measured as a function of decreasing *T*. All AC susceptibility data were acquired with the AC field parallel to the DC field at an excitation amplitude *h*_ac_ from 0.01 to 2 Oe and a frequency of 8 Hz. Test measurements of AC susceptibility at frequencies between 1 and 800 Hz showed that the results were independent of frequency. The superconducting fraction was found as *f* = (1 − *N*)4π|d*M*/d*H*|/*V*, where *N* was the demagnetization factor and *V* the sample's volume. This yielded *f* = 1, that is, all of the authors′ crystals were 100% superconducting.

The superconducting coherence length, ξ, and magnetic field penetration depth, λ, were found from the measured critical fields *H*_c1_ and *H*_c2_ using the standard expressions^[^ ^46^ ^]^ *H*_c2_ = *Φ*_0_/2*πξ* ^2^ and *H*_c1_ = (*Φ*_0_/4*πλ* ^2^)[lnκ  + α(κ)], where $\alpha (\kappa )\;\;=\;\;0.5+(1+\ln2)/(2\kappa -\sqrt{2}+2)$. The GL parameter, κ, was evaluated at all measurement temperatures which showed that it reduced from κ(2*K* = 0.3*T*_c_) = 1.1 to κ(*T*_c_) = 0.75. The critical field for surface superconductivity, *H*_c3_, was determined from AC susceptibility curves such as shown in Figure 1c and Figure S3a, Supporting Information. It was defined as the field *H* corresponding to 0.5% of the χ′ value in the Meissner state. For all of the authors′ crystals, *H*_c3_ = 2*H*_c2_ was obtained at the lowest measurement temperature, *T* = 2K (Figure 2d), in agreement with theory for clean superconductors.^[^ ^46^ ^]^ At higher temperatures, the *H*_c3_/*H*_c2_ ratio gradually decreased, approaching 1.69 close to *T*_c_, again in agreement with expectations.^[^ ^32^ ^]^

## Conflict of Interest

The authors declare no conflict of interest.

## Supporting information

Supporting Information

## Data Availability

The data that support the findings of this study are available from the corresponding author upon reasonable request.
