# Supplementary material for: Magnetization Signature of Topological Surface States in a Non‐Symmorphic Superconductor
Source: Adv Mater. 2021 Aug 8;33(39):2103257. doi: 10.1002/adma.202103257 (PMC11468291; doi:10.1002/adma.202103257)
Supplement: Supplementary file 1 — Supporting Information [file ADMA-33-2103257-s001.pdf]

© 2021 Wiley-VCH GmbH

ADVANCED MATERIALS

Supporting Information

for *Adv. Mater.,* DOI: 10.1002/adma.202103257

Magnetization Signature of Topological Surface States in a Non-Symmorphic Superconductor

Wenjun Kuang, Guillermo Lopez-Polin, Hyungjun Lee, Francisco Guinea, George Whitehead, Ivan Timokhin, Alexey I. Berdyugin, Roshan Krishna Kumar, Oleg V. Yazyev, Niels Walet, Alessandro Principi, Andre K. Geim, and Irina V. Grigorieva**

SUPPORTING INFORMATION

for

Magnetization Signature of Topological Surface States in a Non-Symmorphic Superconductor

Wenjun Kuang, Guillermo Lopez-Polin, Hyungjun Lee, Francisco Guinea, George Whitehead, Ivan Timokhin, Alexey I. Berdyugin, Roshan Krishna Kumar, Oleg Yazyev, Niels Walet, Alessandro Principi*, Andre K. Geim, Irina V. Grigorieva*

1. Structural characterization of In_2Bi crystals.

Prior to collecting the Bragg reflections as described in Methods, a pre-experiment was performed to determine the unit cell and orientation matrix for the crystal. During the pre-experiment, reflections were collected and indexed for a range of crystal orientations, giving both the unit cell of the crystal and the orientation matrix that relates the unit cell axes to the instrument axes. Sharp reflections were observed, indicative of a single crystal (Figure S1a). The observed reflections from the pre-experiment data were indexed to a unit cell with a primitive hexagonal Bravais lattice *a* = 5.471(6) Å, *c* = 6.515(16) Å, *V*=168.9(5) Å^3, indexing against 62 out of 64 observed peaks. This was then used to collect 100% of the unique reflections that were re-indexed to give a primitive hexagonal unit cell of *a* = 5.4728(8) Å, *c* = 6.5333(12) Å, V=169.47(5) Å, in excellent agreement with the unit cell reported for In_2Bi in literature [1]: *a* = 5.4760 Å, *c* = 6.5400 Å, V=169.84 Å^3 (at $T = 195$ K).

Additionally, several single crystals were mechanically flattened to turn them into polycrystals and checked for possible presence of a second phase in powder diffraction mode. Typical spectra are shown in Figure S1b and c. The database search was performed against the extracted diffraction patterns using Panalytical X'pert HighScore Plus to index the peaks. This showed excellent correlation with literature for In_2Bi (ref. [2]). No other phases could be detected.

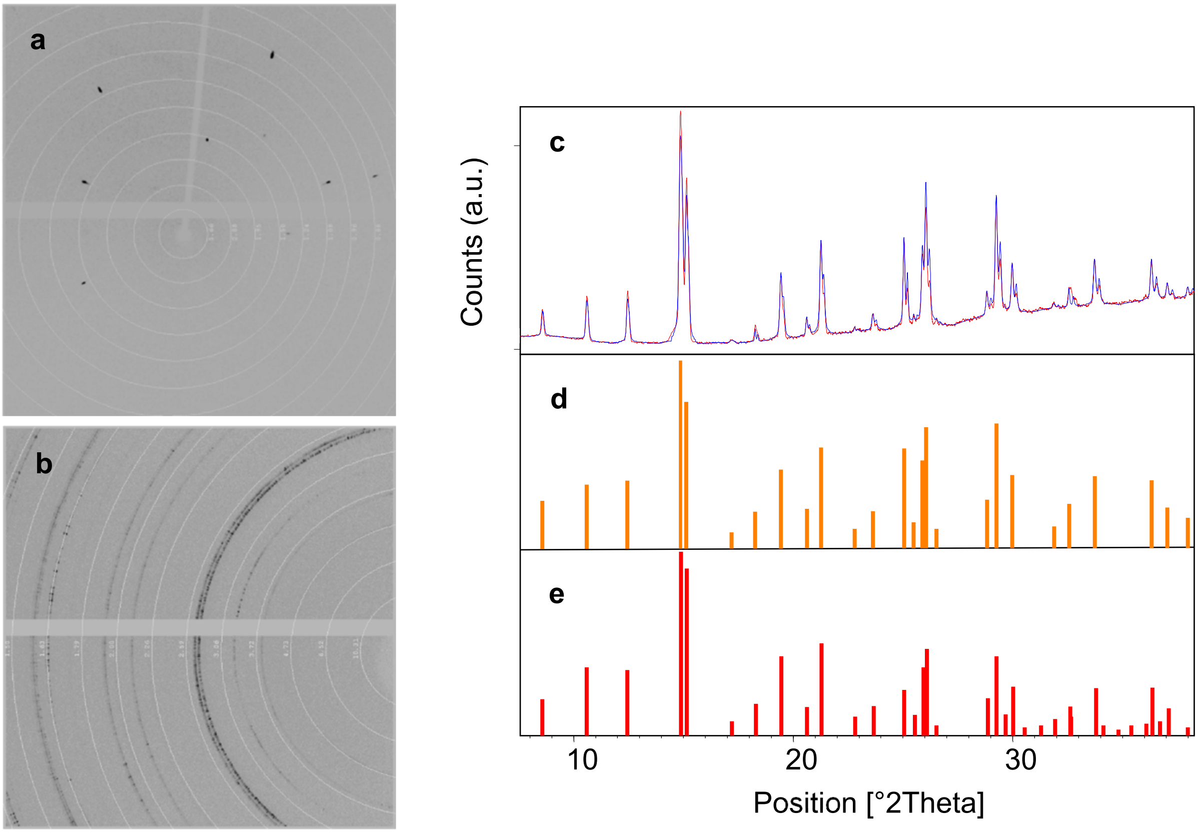

Figure S1 | Structural characterization of our In_2Bi crystals. a,b, Representative frames from the collected XRD data for an as-grown spherical In_2Bi single crystal (**a**) and a polycrystal (mechanically flattened spherical crystal) (**b**). Sharp spots in (**a**) correspond to Bragg reflections for a given orientation of the crystal. Shown are the resolution arcs in Å determined from the distance to the detector and the wavelength used. **c,** Comparison of a measured powder diffraction spectrum for our In_2Bi polycrystal (red line) with a calculated spectrum (blue), indexed and fitted against the database peak positions for In_2Bi. **d,e**, Stick representation on the peak positions and relative intensities of the peaks comparing our collected data (**d**) and published data (**e**).

2. Evidence of In_2Bi oxidation in air and the importance of surface protection.

All magnetisation data in the main text and structural data above were obtained on crystals grown in high vacuum and handled either in the inert (argon) atmosphere of a glove box or immersed in paraffin oil (the latter is known to prevent exposure to oxygen and moisture). This was necessary because an exposure to ambient atmosphere resulted in the appearance of new phases (InBi and In_5Bi_3) at the surface of the crystals. The presence of In_5Bi_3 and small amounts of InBi is evident from XRD spectra for samples exposed to air (Fig. S2a) and from the appearance of a second superconducting phase with $T_c \approx 4.2\text{K}$ in magnetization measurements (Fig. S2b). The above T_c corresponds to the known superconducting transition for In_5Bi_3 (ref. [3]). The fact that second-phase peaks in XRD spectra are relatively high in intensity compared to the In_2Bi host is due to the small penetration depth for Cu-Kα X rays, of the order of few μm. From the ratio of the diamagnetic signals corresponding to the superconducting transitions for In_5Bi_3 and In_2Bi, we estimate the thickness of the In_5Bi_3 layer formed at the surface of In_2Bi after several weeks of exposure to air to be around 2 μm (InBi is not

superconducting under ambient pressure and therefore does not show up in magnetization measurements).

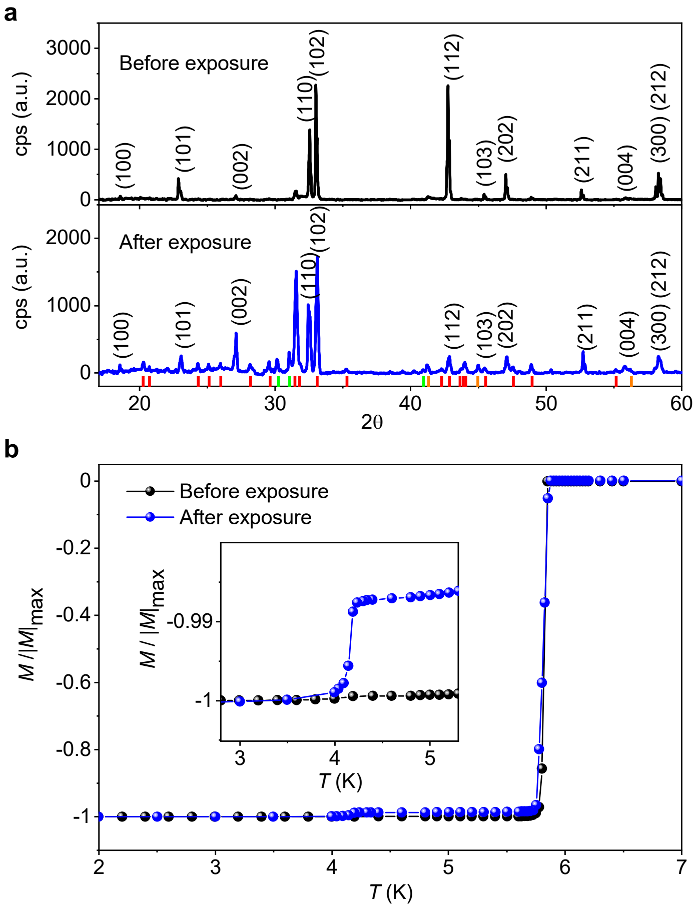

Figure S2 | Importance of surface protection and evidence of In_2Bi oxidation in air. a, XRD spectra before and after exposure to air for 2 days. All peaks corresponding to In_2Bi are labelled. No second phases could be detected in crystals kept in vacuum or in moisture- and oxygen-free environment of a glovebox. Red, green and orange markers correspond to peak positions for In_5Bi_3 (red), InBi (green) or to both phases (orange). XRD data were collected using Rigaku Smartlab diffractometer with Cu Kα radiation (λ=1.5418 Å). **B**, Normalised T-dependent magnetization of an In_2Bi crystal before (black) and after (blue) exposure to air for several weeks. The inset shows a zoomed-up part of the $M(T)$ curves around the expected superconducting transition for In_5Bi_3 ($T_c \approx 4.2\text{K}$).

A likely reason for the observed formation of In_5Bi_3 and InBi is the different enthalpies of oxidation for In and Bi: at room temperature the enthalpy of oxide formation for Bi is H_{Bi2O3} = -575 kJ/mol and for In it is H_{In2O3} = -924 kJ/mol, that is, indium is oxidized more easily. In turn, formation of In_2O_3 at the surface leads to In deficiency, favouring formation of In_5Bi_3 or/and InBi. The In_2O_3 grown at the surface is likely to be amorphous and therefore does not produce any peaks in XRD spectra.

3. Magnetization of spherical single crystals and effect of bulk pinning.

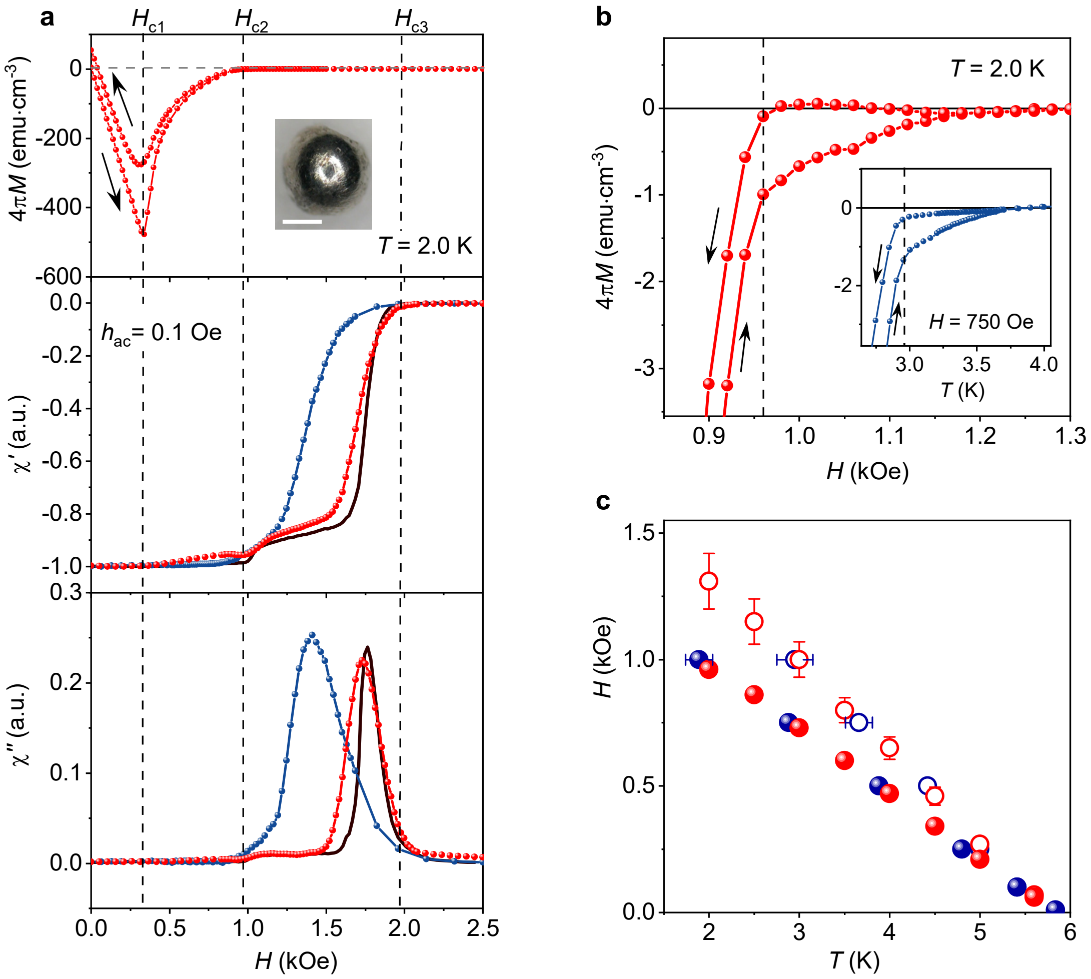

Figure S3| Magnetic response of spherical In₂Bi crystals. a, Typical field-dependent dc magnetisation $M(H)$ of In_2Bi spheres (top panel) and corresponding ac susceptibility (middle and bottom panels, symbols). Shown are measurements at temperature $T = 2$ K and ac field amplitude $h_0 = 0.1$ Oe. For comparison, also shown are data for the cylindrical sample of Fig. 1c in the main text (black lines). To demonstrate the effect of surface degradation, we show ac susceptibility of a spherical crystal before and after its surface was intentionally degraded (sandpapered), red and blue symbols, respectively. The inset in the top panel shows a photo of the as-grown crystal; scale bar 1 mm. **b,** *Main panel*: Hysteresis in field-dependent dc magnetisation, $M(H)$, above the bulk transition to the normal state; temperature $T = 2\text{K}$. Up/down arrows indicate measurements in increasing /decreasing external field. *Inset*: Hysteresis in temperature-dependent magnetisation $M(T)$ measured at $H = 750$ Oe. Up/down arrows correspond to zero-field cooling (ZFC)/field cooling (FC), respectively. Vertical dashed lines indicate the field H or temperature T corresponding to the bulk transition to the normal state. **c,** Phase diagram for the In_2Bi sphere from **b.** Solid red symbols correspond to the bulk transition to the normal state, $H_{c2}(T)$, and solid blue symbols to $T_c(H)$. Open symbols correspond to the disappearance of the hysteresis in $M(H)$ (red) and in $M(T)$, blue. The agreement between the two types of measurements validates the common origin of the diamagnetic surface contribution.

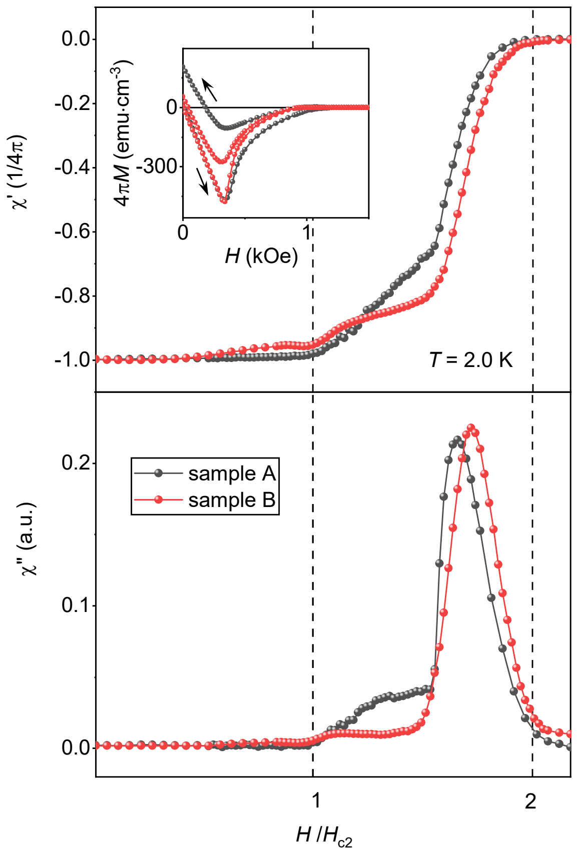

Figure S4 | Effect of bulk pinning on the diamagnetic response of the surface sheath and the transition at H_{ts}. Shown are data for two spherical crystals with different bulk pinning strengths. Stronger bulk pinning for sample A is indicated by a larger hysteresis in dc magnetization $M(H)$ between increasing and decreasing H (indicated by arrows in the inset of the top panel) and a larger remnant M at zero H. The transition at H_{ts} in ac susceptibility is clearly visible but the diamagnetic screening below H_{ts} is weaker compared to sample B (data for sample B also shown in Fig. S3). Bulk pinning is an indication of non-uniformities and the presence of defects in a crystal which typically results in local variations of the superconducting coherence length. To some extent it can be expected to affect the near-surface of the crystal, too, weakening its diamagnetic response [25].

4. Temperature dependence of H_{c2}: fitting to the multiband theory.

To analyse the experimental temperature dependence of H_{c2} we use the two-band model proposed by Gurevich *et al* [4,5]. As discussed in literature [6], the more sensitive indicator of the multiband nature of superconductivity is a strong temperature dependence of the slope of dc magnetisation $\frac{dM}{dH}|_{H=H_{c2}}$, as we indeed observe for In_2Bi. Contributions from multiple bands also modify the temperature dependence of H_{c2}. The model [4,5] takes into account multiple scattering channels that are included via intraband- and interband electron-phonon coupling parameters, λ_{11}, λ_{22} and λ_{21}, λ_{12}, respectively, and normal state electronic diffusivity tensors, $D_m^{\alpha\beta}$, reflecting the underlying symmetry and anisotropy of the Fermi surfaces [4]. An anomalous T dependence of H_{c2} (enhancement at low T) results from different diffusivities for different electronic bands: In refs. [4-6] the model was compared with the well-known example of two-band superconductivity in MgB_2 where the principal diffusivity value $D_\sigma^{(c)}$ along the c axis is much smaller than the two in-plane values $D_\sigma^{(a)}$ and $D_\sigma^{(b)}$ due to the

nearly 2D nature of the σ band (band 1). In contrast, for the 3D π band (band 2), the difference in principal values $D_\pi^{(a)}$, $D_\pi^{(b)}$, and $D_\pi^{(c)}$ is less pronounced, resulting in a disparity of intraband diffusivities D_1 and D_2. From the band structure and Fermi surface topology (Figs 4a, S6, S8), the situation is similar for our In_2Bi where the diffusivity for the electronic states associated with hexagonal In_1Bi_1 planes can be expected to be different from that for the electronic states having a 3D character. This qualitative picture is born out in the observed strong temperature dependence of the slope of dc magnetisation $\frac{dM}{dH}|_{H=H_{c2}}$ described by the Maki parameter κ_2 (Fig. 4b) and its ratio to the GL parameter $\kappa_{GL} = \lambda/\xi$. It follows from our measurements that both κ_{GL} and κ_2 for In_2Bi are temperature dependent, with $\kappa_2 = 0.75 = \kappa_{GL}$ near T_c, as expected [6], and their ratio increasing to $\kappa_2/\kappa_{GL} \approx 2$ at our lowest measurement temperature, 2K (inset in Fig. 4b in the main text). According to analysis of ref. [6], such a large increase corresponds to a diffusivity ratio for different bands $\eta{\sim}0.1$, in agreement with the best fit to our experimental $H_{c2}(T)$ (see Fig. S5) obtained using an implicit expression [4,5]

$$a_0[\ln(t) + U(h)][\ln(t) + U(\eta h)] + a_1[\ln(t) + U(h)] + a_2[\ln(t) + U(\eta h)] = 0 \qquad (1)$$

where $U(x) = \psi(x + 1/2) - \psi(1/2)$, $\psi(x)$ is the digamma function; $a_0 = 2w/\lambda_0$, $a_1 = 1 + \lambda_-/\lambda_0$, $a_2 = 1 - \lambda_-/\lambda_0$, $\lambda_- = \lambda_{11} - \lambda_{22}$, $w = \lambda_{11}\lambda_{22} - \lambda_{12}\lambda_{21}$, $\lambda_0 = (\lambda_-{}^2 + 4\lambda_{12}\lambda_{21})^{1/2}$, $\eta = D_2/D_1$, $t = T/T_c$, and $h = H_{c2}D_1/2\phi_0 T$. To obtain the best fit, we set the diffusivity ratio η as a fitting parameter and tested different sets of coupling parameters λ. This showed that the fit is very sensitive to η, with the best fit corresponding to $\eta = 0.1$ (Fig. S5), i.e., the same value as inferred from the T dependence of $M(H)$ at H_{c2}. In contrast, the fit was found to be practically insensitive to the coupling constants λ_{ii} and λ_{ij} in the available temperature range (inset in Fig. S5) indicating that H_{c2} data alone are insufficient to derive information about electron-phonon couplings in different bands. Importantly, this has no bearing on our discussion and/or conclusions on the role of the topological surface states.

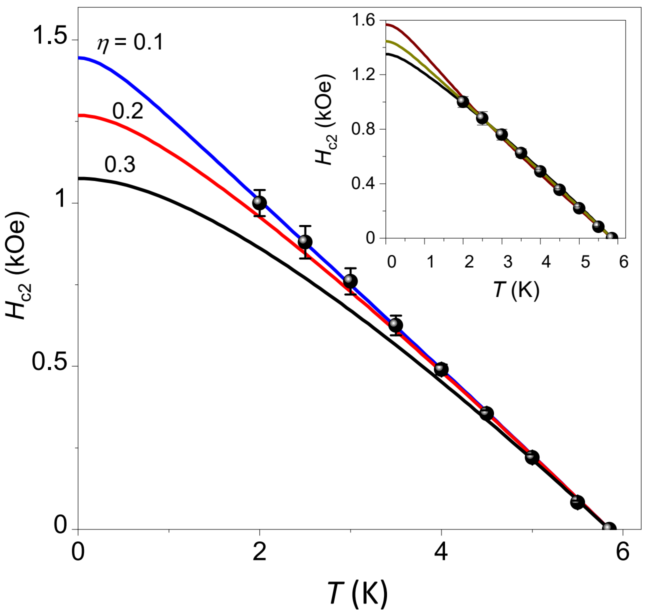

Figure S5 | Temperature dependence of the upper critical field. Shown are $H_{c2}(T)$ data for the cylindrical single crystal of Fig. 2d in the main text. *Main panel:* Solid lines: fits to eq. (1) for the same set of λ_{ij} ($\lambda_{11} = 0.7$; $\lambda_{22} = 0.3$; $\lambda_{12} = \lambda_{21} = 0.8$) and different diffusivity ratios shown as labels. *Inset:* Fits to eq. (1) for $\eta = 0.1$ and three different sets of λ_{ij}. Top curve: $\lambda_{11} = 0.6$; $\lambda_{22} = 0.4$; $\lambda_{12} =$

$\lambda_{21} = 0.6$; middle curve: $\lambda_{11} = 0.8; \lambda_{22} = 0.2; \lambda_{12} = \lambda_{21} = 0.9$; bottom curve: $\lambda_{11} = 0.7; \lambda_{22} = 0.3; \lambda_{12} = \lambda_{21} = 0.8$. In the temperature range where data are available, the fit is equally good for all sets of λ_{ij}. Fits to $H_{c2}(T)$ for all our crystals (cylindrical and spherical) produced similar results.

As expected [4-6], the extrapolated value of $H_{c2}(0) \approx$1.5 kOe (Fig. S5) is considerably higher than the universal value for a single-band superconductor with H_{c2} limited by orbital pair breaking [7], $H_{c2}^{\mathrm{orb}}(0) \approx -0.693T_c(\frac{dH_{c2}}{dT})|_{T=T_c}$. For our crystals with T_c = 5.9 K and $\frac{dH_{c2}}{dT}|_{T=T_c} \approx 220$ Oe/K, $H_{c2}^{\mathrm{orb}}(0) \approx$0.9 kOe.

5. DFT analysis

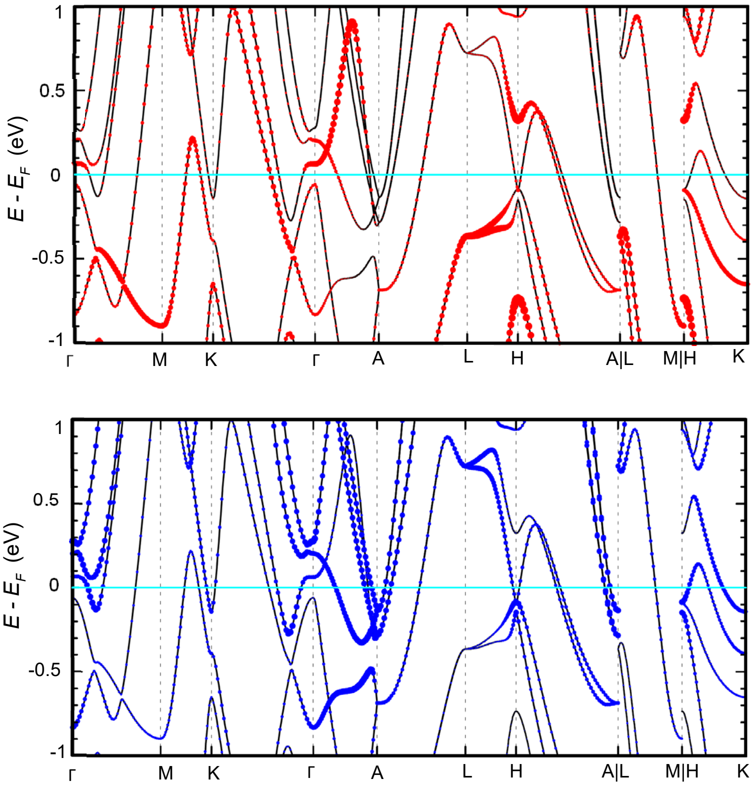

Figure S6 | Band structure of In_2Bi. Bulk band structure calculated using density functional theory. The size of red (blue) dots indicates *p*-orbital contributions to different bands from Bi (top panel) and In (bottom panel) atoms.

Our first-principles electronic structure calculations are based on density functional theory (DFT) [8,9] as implemented in the Vienna *ab initio* simulation package (VASP) [10,11] and a generalized gradient approximation of Perdew-Burke-Ernzerhof-type [12] is employed for the exchange-correlation energy. The electron-ion interaction is described by the projector augmented-wave (PAW) method [13] and the *d* semi-core states are included in the In and Bi PAW datasets used. The spin-orbit coupling is included in all calculations. Wave functions are expanded in terms of plane waves with a kinetic energy cutoff of 400 eV. The ground-state charge density is evaluated on a 32 x 32 x 24 *k*-point mesh. The plane-wave cut-offs and *k*-point meshes are chosen to ensure the convergence of total energies within 0.5 meV. The *ab initio* tight-

binding Hamiltonian is constructed using maximally-localized Wannier functions [14] as a basis, and s and p orbitals centred on In and Bi atoms are used as projection orbitals. In order to preserve the symmetry of the resulting Wannier functions as much as possible, an iterative minimization step was avoided. The resulting Hamiltonian was then used to calculate momentum-resolved density of states using the iterative Green's function method [15]. The DFT results, highlighting the contributions from Bi and In p-orbitals, are shown in Fig. S6.

6. Tight-binding calculations

Looking at the crystal structure of In_2Bi (Fig. 1a of the main text), we recognise In_1Bi_1 honeycomb planes arranged in an AA' configuration (In atoms on top of Bi atoms, and vice versa) and 1D In chains passing through the hexagon centres. The Bi-Bi and In-In distance in the In_1Bi_1 planes are assumed to be the same and equal to a parameter a. The distance between consecutive In_1Bi_1 planes, as well as between the In atoms within the 1D chains is given by $c/2$. The symmetric unit cell (shaded region in Fig. 1a) contains six atoms: two In atoms from the vertical chains, and two In and two Bi atoms from In_1Bi_1 planes. Its height is c. The In (Bi) atom of one layer is mapped onto the In (Bi) atom of the other layer by a screw transformation. This comprises a translation by $c/2$ in the vertical direction, which maps the In (Bi) atom of one layer into the Bi (In) of the other, followed by $180°$ rotation with respect to the vertical axis passing through the midpoint of the cell. The latter transformation maps the In (Bi) atom onto the Bi (In) atom of the same In_1Bi_1 layer. Both operations separately leave the In-wire subsystem invariant. Therefore, their combination leaves the whole system (In_1Bi_1 planes and In chains) invariant. The crystal also exhibits a 3-fold rotational (C_3) axis passing through the centre of an In_1Bi_1 hexagon, which also serves as a 6-fold rotational screw-symmetry axis (C_6), when combined with the screw symmetry above.

Based on the crystallographic considerations, we can construct a minimal tight-binding model that captures the salient features of the In_2Bi band structure and allows us to gain insight into the *ab-initio* DFT results (Fig. S6). As in the DFT calculations, we use the electronic configurations of In and Bi, $[Kr]4d^{10}5s^2 5p^1$ and $[Xe]4f^{14}5d^{10}6s^2 6p^3$, respectively. Accordingly, the In electrons that contribute most to the properties of the compound are those in 5s and 5p orbitals and the contribution of Bi atoms is dominated by 6s and 6p electrons. Furthermore, Bi is a heavier element and has a much stronger spin-orbit interaction. To keep our analysis as simple as possible, we include only one p-like orbital per atom. This is sufficient to reproduce the main features of the band structure around the H (and H') point of the Brillouin zone (Fig. S8a). Some details of the DFT calculations (e.g., the 12 bands crossing the Fermi surface) do not appear in the simple model and would require finer details of orbital hybridization to be included in the tight-binding analysis. Such details are not essential for our purpose here, as the simple model is already capable of explaining the occurrence of topological surface states (see the following section 'Topological surface states').

Our tight-binding model is shown schematically in Fig. S7. To calculate the electronic bands corresponding to the hexagonal In_1Bi_1 planes, we express all parameters in terms of the intralayer hopping between In and Bi atoms, which we call t. The on-site energy of a Bi (In) atom is $+U_0$ ($-U_0$). Keeping in mind that relativistic corrections play an important role, we introduce next-nearest-neighbour Kane-Mele spin-orbit couplings $t_\alpha^{(2)}$, where α = Bi, In (because of their atomic weights, the two atoms are expected to exhibit very different spin-orbit interactions). We use $t_\perp^{(1)}$ to denote the interlayer hopping parameter that couples an In (Bi) atom with the Bi (In) directly above. Similarly, $t_{\perp,\alpha}^{(2)}$ denotes the In-In and Bi-Bi interlayer hopping parameter. The Hamiltonian describing In_1Bi_1 layers is therefore:

$$H_{\mathrm{InBi}} = U_0 \sum_{i,l,\alpha} \sigma^z_{\alpha\alpha}\, c^+_{il\alpha} c_{il\alpha} + t \sum_{\langle i,j\rangle,l,\alpha,\alpha'} c^+_{il\alpha} \sigma^x_{\alpha\alpha'} c_{jl\alpha'} + i \sum_{\langle\langle i,j\rangle\rangle,l,\alpha} t^{(2)}_\alpha \sigma^z_{\alpha\alpha} s^z_{\alpha\alpha} c^+_{il\alpha} c_{jl\alpha}$$
$$+ t^{(1)}_\perp \sum_{\langle l,l'\rangle,i,\alpha,\alpha'} c^+_{il\alpha} \sigma^x_{\alpha\alpha'} c_{il'\alpha'} + \sum_{\langle\langle il,jl'\rangle\rangle,\alpha} t^{(2)}_{\perp,\alpha} c^+_{il\alpha} c_{jl'\alpha},$$

where $c_{il\alpha}$ ($c^+_{il\alpha}$) destroys (creates) an electron in the α = In, Bi atom at position i of the l-th In_1Bi_1 layer, $\sigma^x_{\alpha\alpha'}, \sigma^y_{\alpha\alpha'}, \sigma^z_{\alpha\alpha'}$ ($s^x_{\alpha\alpha'}, s^y_{\alpha\alpha'}, s^z_{\alpha\alpha'}$) are Pauli matrices acting on the sublattice (spin) degree of freedom, while $\langle i,j\rangle$ and $\langle\langle i,j\rangle\rangle$ denote that the summation is restricted to nearest- and next-nearest-neighbour atoms. Finally, the sum over $\langle\langle il,jl'\rangle\rangle$ runs over next-nearest-neighbour atomic sites on adjacent layers.

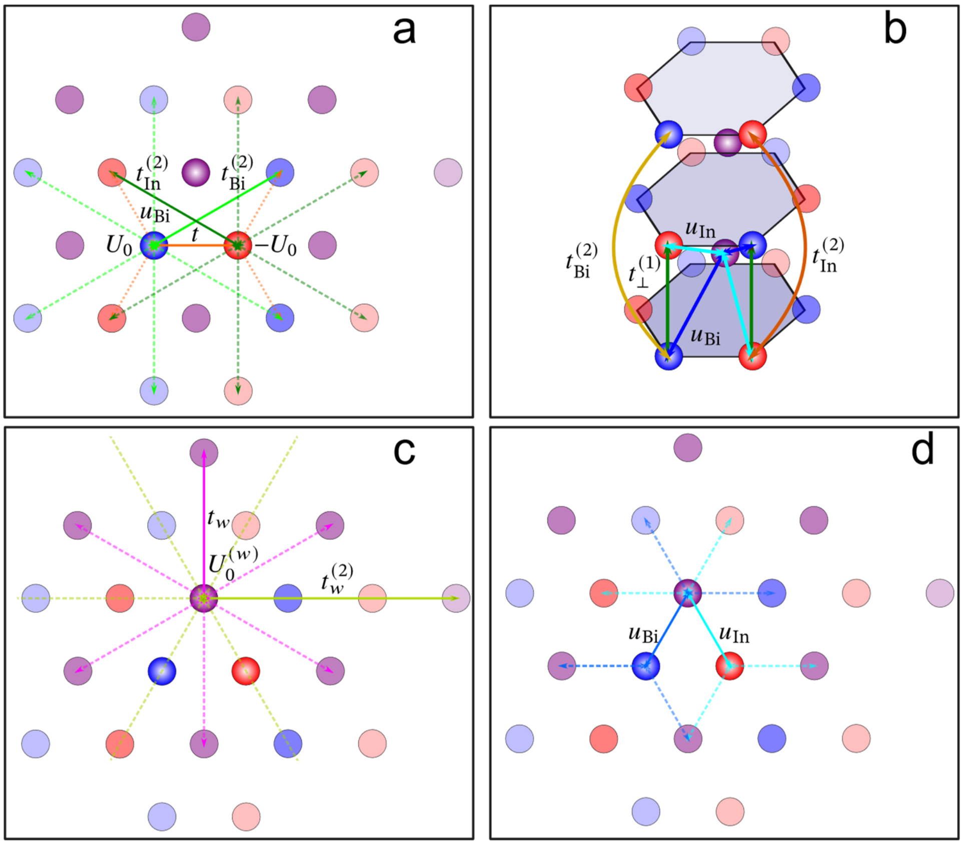

Figure S7: The structure of our tight-binding model. Hamiltonians H_{InBi} (**a**), $H_{InBi-In}$ (**b**, **d**) and H_{In} (**c**). The atoms shown in 3D are those belonging to the symmetric unit cell; **a**,**c**,**d** are top views, and **b** is a side view. The purple atoms represent the In chains' atoms at height $c/4$ and $3c/4$ from the bottom In_1Bi_1 layer of the unit cell. The red (blue) circle stand for In (Bi) atom of the bottom In_1Bi_1 layer and a Bi (In) atom of the In_1Bi_1 layer at height $c/2$.

The vertical In chains are described with an on-site energy $U^{(w)}_0$ and a nearest-neighbour (next-nearest-neighbour) hopping t_w ($t^{(2)}_w$) in the vertical direction. Their Hamiltonian reads

$$H_{\mathrm{In}} = U^{(w)}_0 \sum_{a,\lambda} d^+_{a\lambda} d_{a\lambda} + t_w \sum_{\langle\lambda,\lambda'\rangle,a} d^+_{a\lambda} d_{a\lambda'} + t^{(2)}_w \sum_{\langle\langle\lambda,\lambda'\rangle\rangle,a} d^+_{a\lambda} d_{a\lambda'},$$

where $d_{a\lambda}$ ($d^+_{a\lambda}$) destroys (creates) an electron in the λ-th atom of the In wire at in-plane position a. Finally, we couple the In chains to In_1Bi_1 planes via the hopping parameters u_α (α = In, Bi).

$$H_{\mathrm{InBi-In}} = \sum_{\langle il\alpha,\lambda a\rangle} u_\alpha (d^+_{a\lambda} c_{il\alpha} + c^+_{il\alpha} d_{a\lambda}).$$

Here $\langle il\alpha, \lambda a\rangle$ restricts the sum to nearest-neighbour atoms belonging to an In_1Bi_1 hexagon and an In 'chain'. The full Hamiltonian of the system is $H = H_{\mathrm{InBi}} + H_{\mathrm{In}} + H_{\mathrm{InBi-In}}$.

The band structure is obtained by diagonalizing the combined Hamiltonian, $H_{\mathrm{InBi}} + H_{\mathrm{In}} + H_{\mathrm{InBi-In}}$. The eigenvalues have a complicated analytical form that is not reported here. A representative band structure is shown in Fig. S8b. To obtain this result, we have fitted the tight-binding parameters to the DFT results (Fig. S6) aiming to reproduce the band structure at high-symmetry points of the Brillouin zone. We find $t = 0.45$ eV, $U_0 = 0.4$ eV, $t^{(2)}_{Bi} = 0.05$ eV, $t^{(1)}_{\perp} = -0.65$ eV, $t^{(2)}_{\perp,In} = t^{(2)}_{\perp,Bi} = 0.4$ eV, $U^{(w)}_0 = 0.55$ eV, $t_w = 0.6$ eV, $t^{(2)}_w = -0.2$ eV, $u_1 = u_2 = 0.05$ eV. In the resulting spectrum (Fig. S8b) one recognises a series of band crossings along the $\Gamma - A$ line, two-fold degenerate bands in the $A - L$ direction (forming a "nodal" line) and high-density-of-states bands near Γ and H (H') points in the Brillouin zone. These features are a direct consequence of the In_2Bi nonsymmorphic crystal symmetry. The degeneracy of the Dirac crossings and nodal lines is in fact a consequence of the different transformation properties of the states under C_2 screw-symmetry [16,17]. Since the crystal potential respects such symmetries, the coupling between those states must vanish, no gap can be opened and they remain degenerate. Equivalently, because of the C_2 screw-symmetry, the vertical coupling between In_1Bi_1 planes must vanish at $k_z = \pi/c$, so that they represent two copies of an "asymmetric" Kane-Mele model. Such decoupling explains the twofold degeneracy of the bands along the nodal $A - L$ line. In turn, the symmetry-protected band crossings and nodal lines imply the existence of surface states in the normal state of In_2Bi [18-20]. Those are discussed in the next section.

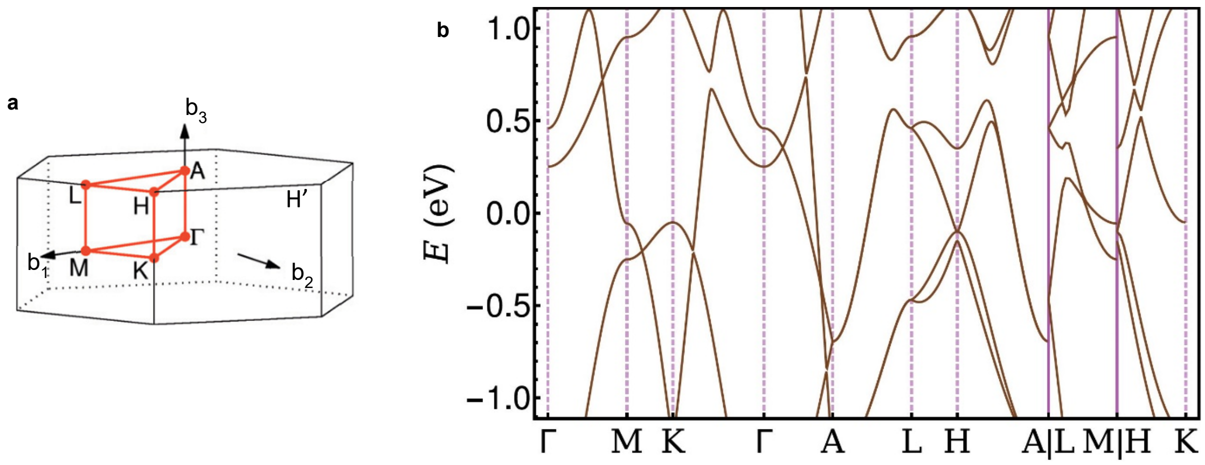

Figure S8 | Band structure of In_2Bi. a, Brillouin zone of In_2Bi. **b,** Bulk band structure calculated using a tight-binding model fitted to *ab-initio* DFT simulations shown in Fig. S6. Positions of high-symmetry points in the Brillouin zone are shown in **a**.

Furthermore, the coupling between In_1Bi_1 planes and In chains is small, too, as can be seen from the values of $u_{1,2}$, suggesting that the two sub-systems contribute nearly independently to the overall response of In_2Bi. The electronic states representing In_1Bi_1 planes and In chains have different dimensionalities. The states at H (H') localised in In_1Bi_1 planes have a pure 2D character, while those associated with In chains

are more of a 3D character. Superconductivity in such a system can therefore be expected to exhibit a coexistence of two weakly coupled superconducting gaps with different dimensionalities, in agreement with the experimental observations.

7. Topological surface states

Surface states in In_2Bi stem from the nontrivial topology of In_1Bi_1 planes and are protected by the screw symmetry as described in the previous section. To show how such states emerge, we consider a thin film modelled as a stack of In_1Bi_1 layers, finite in one direction (and terminated with zigzag edges) and infinite in the other. We apply periodic boundary conditions in the latter direction. We further simplify the model introduced in section 6 by neglecting all next-nearest-neighbour hopping amplitudes, with the crucial exception of the Kane-Mele-type spin-orbit couplings that are essential for the emergence of topologically protected surface states. We note that the resulting model lacks some of the features of the full one introduced in section 6 (for example, it does not account for the particle-hole asymmetry of the band structure). Such features, arising from the weak coupling between In_1Bi_1 planes and In chains, are not important for describing the surface states.

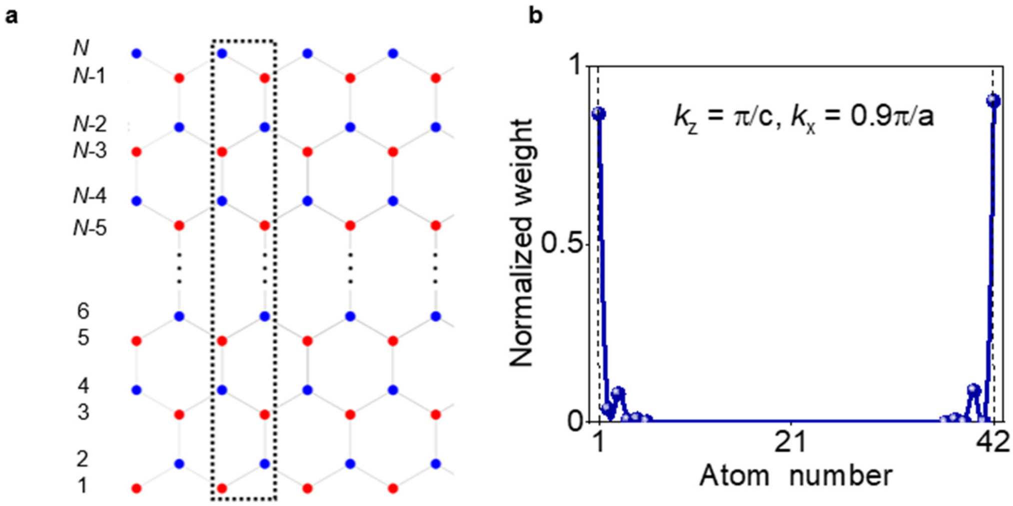

Figure S9 | Spatial localization of topological surface states. a, Schematic of the lattice used in our finite-size calculations. The crystal is modelled as an infinite stack of In_1Bi_1 hexagonal sheets, with In (Bi) aligned on top of Bi (In). Each sheet is terminated with zigzag edges and is infinite in the other direction. The unit cell encompasses two consecutive layers and contains N atoms in each of them (along the finite direction). The dashed line marks the unit cell in one such layer. Periodic boundary conditions are applied in both the in-plane infinite direction, and in the direction perpendicular to the planes. **b,** Normalised weight of the edge state wavefunction on the atoms of one layer of the InBi unit cell (symbols). The edge state has exactly the same weight on the atoms of the other layer. In this calculation, the number of atoms in each layer N=42. Connecting line is a guide to the eye.

The unit cell of In_2Bi (shown in Fig. 1a of the main text) encompasses two consecutive layers and contains two atoms per row $j = 1, \dots, N$, one in each layer. For calculation purposes, it is convenient to introduce units composed by pairs of rows (j and $j+1$) each containing four atoms, one In and one Bi per layer. The In and Bi atoms in the two different rows are distinguished by the sublattice degree of freedom. We

define $\Gamma_{abc} = \tau^a \otimes \sigma^b \otimes s^c$ where τ^a, σ^a and s^a ($a = x, y, z$) are three sets of Pauli matrices operating on the layer, sublattice and spin degrees of freedom, respectively, while $\otimes$ denotes the tensor product. The Hamiltonian operating on the four sites in two consecutive rows of the unit cell is

$$H_{\tilde{j}\tilde{j}} = 2\sin(k_x a)\left(\frac{t_{Bi}^{(2)} + t_{In}^{(2)}}{2}\Gamma_{333} + \frac{t_{Bi}^{(2)} - t_{In}^{(2)}}{2}\Gamma_{303}\right) - U_0\Gamma_{330} + 2t\cos\left(\frac{k_x a}{2}\right)\Gamma_{010} + t_{\perp}^{(1)}\cos\left(\frac{k_z c}{2}\right)\Gamma_{100},$$

whereas the hopping between successive pairs of rows is given by

$$H_{\tilde{j}\tilde{j}+1} = -2\sin\left(\frac{k_x a}{2}\right)\left(\frac{t_{Bi}^{(2)} + t_{In}^{(2)}}{2}\Gamma_{333} + \frac{t_{Bi}^{(2)} - t_{In}^{(2)}}{2}\Gamma_{303}\right) + \frac{t}{2}(\Gamma_{010} + i\Gamma_{020}).$$

In these equations, $\tilde{j} = 1, \dots, N/2$ denotes the pair of rows, while k_x is the quasi-momentum along the in-plane infinite direction. Conversely, k_z is the quasi-momentum in the direction orthogonal to the In_1Bi_1 planes. The other parameters entering these equations and their numerical values are given in section 6.

The full Hamiltonian of the film is obtained by combining $N/2$ $H_{\tilde{j}\tilde{j}}$-blocks along the diagonal of an $4N \times 4N$ matrix. Different pairs of rows are connected by off-diagonal $H_{\tilde{j}\tilde{j}+1}$-blocks. The resulting matrix is diagonalized for fixed k_z and as a function of k_x. Representative results are shown in Fig. 4c in the main text: At $k_z = \pi/c$ two Dirac cones appear from the projection of the bulk band structure onto the surface Brillouin zone. Edge states connect bulk bands and cross the gap, forming a series of linear crossings. This in turn implies that surface states cross the Fermi energy which, according to DFT calculations (section 5) is located around the Dirac points of the bulk band structure. Finally, the weights of the edge states on the atoms of a given layer (Fig. S9b) are obtained by projecting the eigenstates of the $4N \times 4N$ Hamiltonian into given spin and layer components.

8. AC susceptibility and dc magnetization in conventional superconductors: Contribution of surface superconductivity

Magnetization and susceptibility measurements on superconductors detect signals that have their origins in circulating persistent shielding currents [21-26]. The basic idea is that a superconducting surface sheath can support a finite (non-zero) current; as long as this sheath of current is continuous, it will screen the total volume of a superconductor, irrespective of whether its bulk is in normal or mixed state [23-26].

For a superconducting cylinder in a parallel dc magnetic field H_a and a superimposed ac field $h = h_0 \mathrm{e}^{i\omega t}$ ($h \,||\, H_\mathrm{a}$), the susceptibility is given by [21,22]

$$\chi = \frac{1}{4\pi}\left[-1 + \frac{2}{d^2 H_\mathrm{a}}\int_0^d B(r) r dr\right]$$

where $B(r)$ is the magnetic induction inside the sample and d radius of the cylinder. As shown in refs. [21,22], $B(r)$ is the solution of the differential equation

$$\nabla^2 B + K^2 B = 0$$

with the boundary condition $B(d) = H_a$ and K given by

$$K^2 = \frac{2i}{\delta^2}\left(1 - \frac{n_s}{n_0}\right) - \frac{1}{\lambda_L^2} \qquad (2)$$

where $\delta = c/\sqrt{2\pi\sigma\omega}$ is the skin depth related to the electrical conductivity σ, c the speed of light, n_s the density of superconducting electrons, n_0 the total electron density and $\lambda_L = \sqrt{mc^2/4\pi n_s e^2}$ the London penetration depth. The real part of susceptibility χ is then given by [21]

$$\chi' = \mathrm{Re}\{4\pi^{-1}[-1 + 2J_1(Kd)/KdJ_0(Kd)]\} \qquad (3)$$

where J_1 and J_0 are Bessel functions. At low frequencies used in our experiments (all measurements presented in the main text were taken at $\omega/2\pi$=8 Hz) the skin depth is $\delta \gg d \gg \lambda_L$ (δ ~10 cm and $\lambda_L \approx 60$ nm), so that the first term in (2) can be neglected and K^2 becomes

$$K^2 \approx -\frac{1}{\lambda_L^2} = -\frac{4\pi n_s e^2}{mc^2}. \qquad (4)$$

Accordingly, $|Kd|$ in (3) can be replaced with d/λ_L, so that for a given H_a the real part of susceptibility χ' is determined largely by the ratio of the superconductor's size and the magnetic field penetration depth λ_L. At and below H_{c2}, a continuous surface sheath screens the whole interior of the superconductor (as long as κ~1), resulting in perfect diamagnetic screening of small ac fields up to H_{c2}, i.e. $\chi' = -1/4\pi$ [23,24]. Such perfect screening, similar to the Meissner state, is seen for $H < H_{c2}$ in our measurements (Figs 1c and 2a in the main text). At $H_a > H_{c2}$, the order parameter $|\varphi|$ and therefore the superfluid fraction $\overline{n_s} = n_s/n_0$ become gradually suppressed [26], resulting in a gradual decrease of the diamagnetic susceptibility following eq. (2) (recall that $\overline{n_s} = |\varphi|^2$ (ref. [27]). The magnetic field dependence of susceptibility, $\chi'(H_a)$, is then determined by the evolution of the order parameter $|\varphi|^2(H_a)$, or $\overline{n_s}(H_a)$, with the magnetic field.

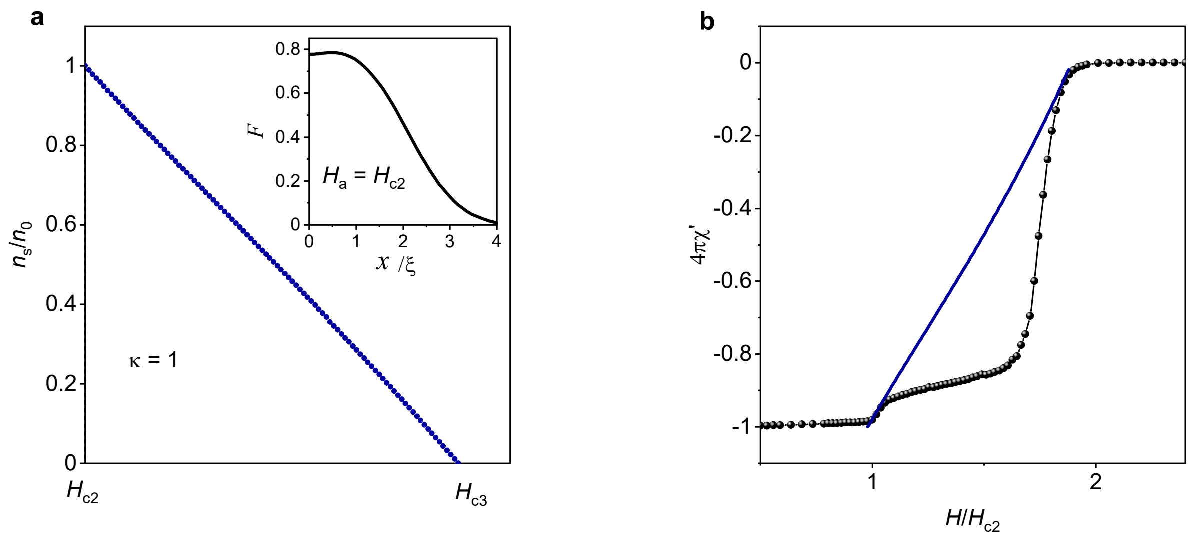

Figure S10 | Magnetic field dependence of the calculated superfluid density and ac susceptibility for a conventional superconductor and comparison with experimental susceptibility for In_2Bi. a, Superfluid fraction in the surface superconducting sheath, n_s/n_0, as a function of the external magnetic field H_a. *Inset*: spatial variation of the normalised order parameter $F(x)$ at $H_a = H_{c2}$ for GL parameter κ=1. The value of the order parameter at the surface, $F(0)$, is determined by requiring that $F(x)$ converges to zero inside the superconductor's bulk ($x \to \infty$). The calculated value of $F(0) \approx 0.8$ reproduces the result of ref. [26]. **b**, Comparison of the experimental ac susceptibility χ' for an In_2Bi cylinder (black symbols, data of Fig. 1c in the main text) with $\chi'(H)$ calculated from n_s/n_0 (H_a) in **a** (blue line).

To compare the expected dependence $\chi'(H_a)$ with our data at $T = 2$K (where $n_s \approx n_0$ at and below H_{c2}), we followed ref. [26] where exact solutions of the Ginzburg-Landau equations were obtained for surface superconductivity as a function of $H_{c2} \le H_a \le H_{c3}$ and κ. First, we calculated numerically the normalised amplitude of the order parameter in the surface sheath, $F(H_a)$, where F is defined as $F(qx) = D(qx)(|D_0|)^{-1}$, $D(qx)$ and D_0 stand for the amplitude of the order parameter at position x in applied field H_a and in zero field, respectively, and $qx = (x/\xi)(H_a/H_{c2})^{1/2}$, see ref. [26] for details. Using the corresponding equations in ref. [26], we found the spatial variation of $F(x/\xi)$ within the surface superconducting layer for different H_a, with the corresponding $\int_0^{4\xi} F^2(t)dt$ proportional to the superfluid fraction $\overline{n_s}$ in the surface sheath. The inset of Fig. S10a shows the result at $H_a = H_{c2}$ (reproducing the calculations in ref. [26]) and the main panel shows the corresponding H dependence of $\overline{n_s}$.

The obtained results for $n_s/n_0(H_a)$ were then used to calculate the susceptibility $\chi'(H_a)$ using (3) and (4). The result is shown in Fig. S10b by the blue line: χ' is expected to decrease approximately linearly as the field increases from H_{c2} to H_{c3}. Such a smooth, near-linear dependence of χ' is in agreement with observations on high-quality Nb in literature (e.g., ref. [28]). A similar smooth $\chi'(H_a)$ dependence was also observed in our In_2Bi samples with a roughened surface and at relatively large amplitudes of the ac field (Figs 3b and 2a, respectively). It is however in stark contrast to our observations for In_2Bi crystals with smooth surfaces at small ac amplitudes, where χ' changes little up to H_{ts} (Fig. S10b).

DC magnetization measurements probe the total magnetic moment in the sample, therefore the contribution from the surface sheath can only be seen at and above H_{c2} (below H_{c2} it is masked by bulk magnetization). As discussed in detail in refs [23,25], for a cylinder of radius R, magnetization per unit volume M and the maximum current that the sheath can sustain are size dependent and inversely proportional to $R^{1/2}$, i.e., the larger the radius, the smaller the current and the magnetic moment, due to the 'cost' in magnetization energy for the whole volume of the cylinder. Nevertheless, M is always much larger [by a factor proportional to $(R/\lambda)^{1/2}$] than the equilibrium moment M_0 corresponding to zero total current in the sheath. As the field is increased above H_{c2}, M decreases due to a decrease in the free energy of the sheath [25].

9. Effect of topological surface states on surface superconductivity

To find how the presence of the topological surface states modifies the properties of the superconducting surface sheath we extend the existing theory of surface superconductivity [26]. The latter yields the spatial distribution of the order parameter $\varphi(\boldsymbol{r})$ and its evolution with the external dc field as it varies between H_{c2} and H_{c3}. As shown in ref. [26] (and reproduced in the inset of Fig. S10a) the order parameter in conventional superconductors remains constant over a distance $x \approx \xi$ from the surface and decays exponentially to zero at $x \approx 4\xi$. At H_{c2} the order parameter at the surface is close to its bulk value but, as the external field increases, it decreases approximately linearly and vanishes at H_{c3}. This result [26] was obtained by solving the Ginzburg-Landau equations with the conventional boundary condition for a superconductor-dielectric interface, that is, that the derivative of the order parameter vanishes at the superconductor's surface [29].

Of particular relevance to our experimental observations is the effect of the superconducting topological surface states on the superfluid density (fraction of the superconducting electrons) $\overline{n_s} = |\varphi|^2$ as a function of the applied dc magnetic field. As shown below, their presence changes the boundary condition, greatly enhancing the overall $\overline{n_s}$ in the surface sheath as compared to the case of conventional

surface superconductivity and concentrating the screening currents in a narrow region at the surface. This results in a much more robust screening of magnetic field above H_{c2}.

Following ref. [26], we consider the Ginzburg-Landau free energy

$$\mathcal{F}[\varphi, \boldsymbol{A}] = \int d\boldsymbol{r} \left\{ -\alpha\, |\varphi(\boldsymbol{r})|^2 + \frac{\beta}{2}\, |\varphi(\boldsymbol{r})|^4 + \frac{[\boldsymbol{\nabla} \times \boldsymbol{A}(\boldsymbol{r})]^2}{8\pi} + \frac{1}{2m^*} \left| -i\hbar \boldsymbol{\nabla}\varphi(\boldsymbol{r}) + \frac{e^*}{c} \boldsymbol{A}(\boldsymbol{r})\varphi(\boldsymbol{r}) \right|^2 \right\},$$

where $\alpha = \hbar^2/(2m^*\xi^2)$, ξ is the coherence length, $\beta = 4\pi\alpha^2/H_c^2$, H_c the thermodynamic critical field, $\varphi(\boldsymbol{r})$ the superconducting order parameter, $\boldsymbol{A}(\boldsymbol{r})$ the vector potential, m^* the particle mass and $e^* = 2e$. Here e is the electron charge. In the Meissner state, the equilibrium order parameter is $\bar{\varphi} = -\alpha/b$. The London penetration length is $\lambda = 4\pi(e^*|\bar{\varphi}|)^2/(m^*c^2)$, and the Ginzburg-Landau parameter $\kappa = \lambda/\xi$.

We consider a planar geometry such that the superconductor occupies the half space $x > 0$, while the topological surface state is assumed to have zero thickness and located at $x = 0$. To derive equations amenable for numerical solution, we rescale the lengths with $\xi\mu$, where $\mu = \sqrt{H_{c2}/H_{\mathrm{dc}}}$, and introduce a dimensionless order parameter $F(\zeta)$ such that $\varphi(\zeta) = \bar{\varphi}\, F(\zeta)e^{ik\varsigma}$. $F(\zeta)$ is a real function, whereas $\zeta = x/\xi\mu$ and $\varsigma = y/\xi\mu$ are dimensionless variables. Similarly, the vector potential is rescaled with $\sqrt{2}\lambda H_c/\mu$. In these equations, H_{dc} is the applied dc magnetic field and $H_{c2} = \sqrt{2}\kappa H_c$.

We further introduce [26] a constant a_0 and the function $a(\zeta)$ to rewrite the dimensionless vector potential as $\zeta + a_0 + a(\zeta)$. Here, the first term is the vector potential due to the applied dc field, and a_0 is the total vector potential at the superconductor's surface. Accordingly, $a(0) = 0$. In terms of these functions, the free energy becomes

$$\mathcal{F}[F, a] = \frac{\xi S H_c^2}{4\pi\mu^2} \int_0^\infty d\zeta \left\{ \mu^2 \left(\frac{F^2(\zeta)}{2} - 1 \right) F^2(\zeta) + \frac{\kappa^2}{\mu^2} \left[1 + \partial_\zeta a(\zeta)\right]^2 + \left[\partial_\zeta F(\zeta)\right]^2 \right.$$
$$\left. + [\zeta - \Gamma + a(\zeta)]^2 F^2(\zeta) \right\},$$

where $\Gamma = k - a_0$ (recall that k is the wavevector that controls the phase of the order parameter) and S is the area of the superconductor's surface. Taking the functional derivative of $\mathcal{F}[F, a]$ with respect to $F(\zeta)$ and $a(\zeta)$ we find the Ginzburg-Landau equations

$$\begin{cases} \partial_\zeta^2 F(\zeta) + \mu^2 F^2(\zeta)[1 - F^2(\zeta)] - [\zeta - \Gamma + a(\zeta)]^2 F(\zeta) = 0 \\ \dfrac{\kappa^2}{\mu^2} \partial_\zeta^2 F(\zeta) = F^2(\zeta)[\zeta - \Gamma + a(\zeta)] \end{cases} \quad (5).$$

These equations must be complemented by the following constraint

$$\Gamma = \mu \sqrt{1 - \frac{F^2(0)}{2} + \left[\partial_\zeta F(\zeta)\right]_{\zeta=0}^2} \quad (6),$$

which is obtained by minimizing the free energy with respect to Γ [26]. By imposing the appropriate boundary conditions at $\zeta = 0$ (see below), Eqs. $(5) - (6)$ are solved to yield $F(\zeta)$ and $a(\zeta)$. In both ref.[26] and the present calculations, $a(0)$ and $\partial_\zeta a(x)|_{\zeta=0}$ are required to vanish at $\zeta = 0$, while $F(\zeta)$ and $a(\zeta)$ must converge to a constant deep in the superconductor's bulk ($\zeta \to \infty$). In particular, $F(\zeta \to \infty) = 0$ (recall that we consider the case of $H_{\mathrm{dc}} > H_{c2}$). In ref. [26], the set of Eqs. $(5) - (6)$ was solved by imposing the conventional (Abrikosov) boundary condition [29] for a superconductor-dielectric interface, which is

$\partial_\zeta F(\zeta)|_{\zeta=0} = 0$. $F(0)$ was then determined with a shooting method so that the function $F(\zeta)$ satisfied all the above requirements.

To reflect the presence of the topological surface states in our experiment, we employ a different boundary condition. Physically, we must require that the total current across the boundary vanishes. This is generally satisfied by imposing [27]

$$\partial_\zeta F(\zeta)|_{\zeta=0} = \frac{F(0)}{b},$$

where b is a constant. For a superconductor-dielectric interface, $b \to \infty$. For a boundary between two superconductors, b can be finite and negative. This should happen if one of the superconductors is a film with thickness much smaller than the magnetic field penetration depth λ and, therefore, its order parameter is little affected by the applied magnetic field [30].

Our experimental system can be thought of as an atomically-thin metallic film of thickness d_{ts}, which surrounds a superconducting cylinder. Accordingly, we model the system as two mutually proximitized superconductors, one corresponding to bulk In_2Bi and the other to its topological surface state. In a magnetic field parallel to the surface, the critical field of the film itself must be strongly enhanced because of its atomic-scale thickness [27] (recall that the thickness of the surface sheath due to the superconducting bulk is relatively large: $d \approx 2\xi > \lambda$, $\xi \approx 60$ nm and $\lambda \approx 65$ nm; see main text). Being so thin, the topological superconducting film is expected to be essentially unaffected by $H_{\mathrm{dc}} < H_{\mathrm{c3}}$, which allows us to assume $F(0) = 1$ for all relevant values of H_{dc}. In turn, this 'pinned' order parameter is found to modify the boundary condition for the surface superconducting sheath associated with the bulk superconductor.

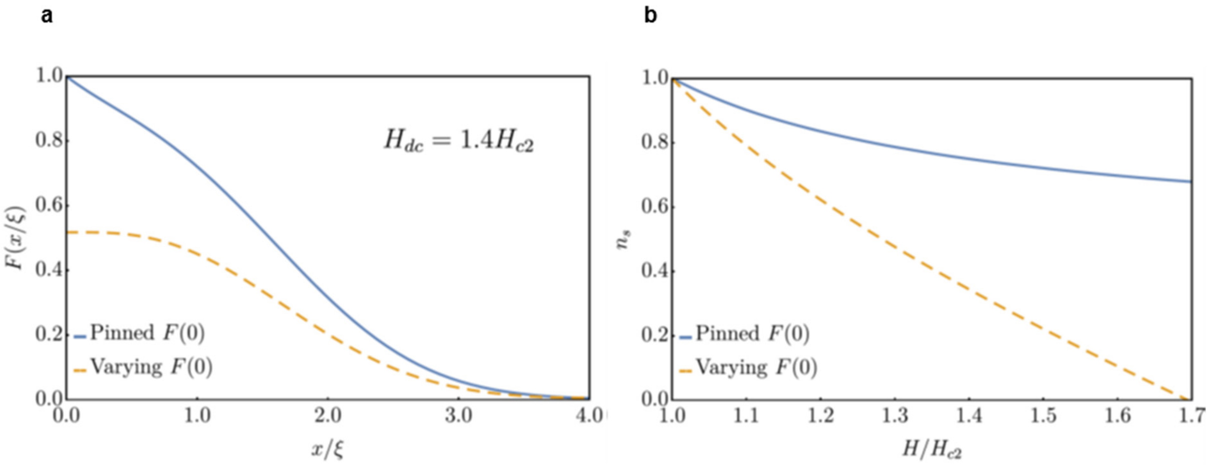

Figure S11 | Solutions of the Ginzburg-Landau equations with and without the superconducting topological state at the surface. a, Comparison between the conventional behaviour [varying $F(0)$] and our case of F 'pinned' to unity at the surface. **b,** The corresponding superfluid densities. In the 'pinned' case, the surface superconductivity is less effected by applied field H.

Based on these considerations, we solve the Ginzburg-Landau equations $(5)-(6)$ by imposing $F(0) = 1$ and determine the resulting b by a shooting method so that $F(\zeta \to \infty) = 0$ and $a(\zeta \to \infty)$ converges to a constant. The resulting order parameter is shown in Fig. S11a. One can see that, for a given field $H_{c2} < H < H_{c3}$, F is considerably enhanced in the whole $\sim 4\xi$ surface layer, compared to the conventional behaviour for the surface superconductivity [26], which is also shown in Fig. S11a. As a

consequence, the superfluid density above H_{c2} , $n_s \propto \int_0^\infty d\zeta\, F^2(\zeta)$, decreases much slower with increasing the magnetic field than in the conventional case [26] (Fig. S11b). Because above H_{c2} the experimentally measured ac susceptibility is determined by $\overline{n_s}(H/H_{c2})$ (see the previous section), the results of Fig. S11b can be translated directly into the susceptibility. The resulting behaviour is plotted in Fig. 4d (main text) showing good agreement between the experiment and theory.

Note that the Ginzburg-Landau free energy is generally reduced if $F(\zeta)$ has a negative slope at $x = 0$. Therefore, it is plausible to argue that the system will always try to minimize its energy by realising an order parameter that peaks at $x = 0$. The presence of the topological surface state opens up such a possibility, and the system readily adapts. For analysis beyond the phenomenological Ginzburg-Landau equations, it would require considering the microscopic interplay between the order parameters in the surface sheath and topological states, which involves self-consistent solution of the Gor'kov equation [29]. This feat is beyond the scope of the present work.

References for Supporting Information

1. R. Kubiak, J. Janczak, *J. Alloys Compd.* **1993**, 196, 117.

2. T. Degen, M. Sadki, E. Bron, U. König, G. Nénert, *Powder Diffraction* **2014**, *29*, 13.

3. Mori, K., Tamura, N., Saito, Y. Superconductivity and electrical resistivity saturation in intermetallic compound In_5Bi_3. *J. Phys. Soc. Jap.* **50**, 1275-1280 (1981).

4. Gurevich, A. Enhancement of the upper critical field by nonmagnetic impurities in dirty two-gap superconductors. *Phys. Rev. B* **67**, 184515 (2003).

5. Gurevich, A. Limits of the upper critical field in dirty two-gap superconductors. *Physica C* **456**, 160-169 (2007).

6. Silaev, M. Magnetic behavior of dirty multioband superconductors near the upper critical field. *Phys. Rev. B* **93**, 214509 (2016).

7. Werthamer, N. R., Helfand, E. & Hohenberg, P. C. Temperature and purity dependence of the superconducting critical field, Hc2. III. Electron spin and spinorbit effects. *Phys. Rev.* **147**, 295–302 (1966).

8. Hohenberg, P. & Kohn, W. Inhomogeneous Electron Gas. *Phys. Rev.* **136**, B864 (1964).

9. Kohn, W. & Sham, L. J. Self-Consistent Equations Including Exchange and Correlation Effects. *Phys. Rev.* **140**, A1133 (1965).

10. Kresse, G. & Hafner, J. Ab initio molecular dynamics for liquid metals. *Phys. Rev. B* **47**, 558(R) (1993).

11. Kresse, G & Furthmüller, Efficient iterative schemes for ab initio total-energy calculations using a plane-wave basis set. *Phys. Rev. B* **54**, 11169 (1996).

12. Perdew, J. P., Burke, K. & Ernzerhof, M. Generalized Gradient Approximation Made Simple. *Phys. Rev. Lett.* **77**, 3865 (1996).

13. Blöchl, P. E. Projector augmented-wave method. *Phys. Rev. B* **50**, 17953 (1994).

14. Mostofi, A. A. et al. An updated version of wannier90: A tool for obtaining maximally-localised Wannier functions. *Comput. Phys. Commun.* **185**, 2309 (2014).

15. López Sancho, M. P., López Sancho, J. M. & Rubio, J. Highly convergent schemes for the calculation of bulk and surface Green functions. *J. Phys. F* **15**, 851 (1985).

16. Zhao, Y. X., Schnyder, A. P. Nonsymmorphic symmetry-required band crossings in topological semimetals. *Phys. Rev. B* **94**, 195109 (2016).

17. Ezawa, M. Hourglass fermion surface states in stacked topological insulators with nonsymmorphic symmetry. *Phys. Rev. B* **94**, 155148 (2016).

18. Wang, Z., Sun, Y., Chen, X.-Q., Franchini, C., Xu, G., Weng, H., Dai, X., Fang, Z. Dirac semimetal and topological phase transitions in A_3Bi (A = Na, K, Rb). *Phys. Rev. B* **85**, 195320 (2012).

19. Yang, B.-J., Bojesen, T. A., Morimoto, T., Furusaki, A. Topological semimetals protected by off-centered symmetries in nonsymmorphic crystals. *Phys. Rev. B* **95**, 075135 (2017).

20. Shiozaki, K., Sato, M., Gomi, K. Topology of nonsymmorphic crystalline insulators and superconductors, *Phys. Rev. B* **93**, 195413 (2016).

21. Khoder, A. F. The superconducting transition and the behavior of the ac susceptibility. *Phys. Lett. A* **94A**, 378-380 (1983).

22. Van de Klundert, L. J. M., Gijsbertse, E. A., Van der Marel, L. C. On the AC susceptibility of metals in the normal or superconducting state. *Physica* **69**, 159-170 (1973).

23. Fink, H. J., Barnes, L. J. Critical state of the superconducting surface sheath. *Phys. Rev. Lett.* **15**, 792-795 (1965).

24. Barnes, L. J., Fink, H. J. Critical currents in the superconducting surface sheath. *Phys. Rev.* **149**, 186-191 (1966).

25. Park, J. G. Persistent currents induced in the superconducting surface sheath. *Adv. Phys.* **18:72**, 103-155 (1969).

26. Fink, H. J., Kessinger, R. D., Exact solutions of the superconducting surface sheath, *Phys. Rev.* **140**, A1937 (1965).

27. Tinkham, M., Introduction to superconductivity. 2nd edition, McGraw-Hill, New York, 1996.

28. Ostenson, J. E., Finnemore, D. K., Critical phenomena in sheath superconductivity of Nb. *Phys. Rev. Lett.* **22**, 188–190 (1969).

29. Abrikosov, A. A., Concerning surface superconductivity in strong magnetic fields. *Sov. Phys. JETP* **20**, 480-488 (1965).

30. Fulde, P. Cooper pair breaking, *Mod. Phys. Lett. B* **24**, 2601 (2010).
